# Perirenal Adipose Tissue Inflammation: Novel Insights Linking Metabolic Dysfunction to Renal Diseases

**DOI:** 10.3389/fendo.2021.707126

**Published:** 2021-08-02

**Authors:** Safaa H. Hammoud, Ibrahim AlZaim, Yusra Al-Dhaheri, Ali H. Eid, Ahmed F. El-Yazbi

**Affiliations:** ^1^Department of Pharmacology and Therapeutics, Faculty of Pharmacy, Beirut Arab University, Beirut, Lebanon; ^2^Department of Pharmacology and Toxicology, Faculty of Medicine, American University of Beirut, Beirut, Lebanon; ^3^Departmment of Biochemistry and Molecular Genetics, Faculty of Medicine, American University of Beirut, Beirut, Lebanon; ^4^Department of Biology, United Arab Emirates University, Al-Ain, United Arab Emirates; ^5^Department of Basic Medical Sciences, College of Medicine, Qatar University, Doha, Qatar; ^6^Biomedical and Pharmaceutical Research Unit, Qatar University (QU) Health, Qatar University, Doha, Qatar; ^7^Department of Pharmacology and Toxicology, Faculty of Pharmacy, Alexandria University, Alexandria, Egypt; ^8^Faculty of Pharmacy, Alalamein International University, Alalamein, Egypt

**Keywords:** perirenal adipose tissue, chronic kidney disease, cardiovascular disease, metabolic dysfunction, adipose tissue inflammation

## Abstract

A healthy adipose tissue (AT) is indispensable to human wellbeing. Among other roles, it contributes to energy homeostasis and provides insulation for internal organs. Adipocytes were previously thought to be a passive store of excess calories, however this view evolved to include an endocrine role. Adipose tissue was shown to synthesize and secrete adipokines that are pertinent to glucose and lipid homeostasis, as well as inflammation. Importantly, the obesity-induced adipose tissue expansion stimulates a plethora of signals capable of triggering an inflammatory response. These inflammatory manifestations of obese AT have been linked to insulin resistance, metabolic syndrome, and type 2 diabetes, and proposed to evoke obesity-induced comorbidities including cardiovascular diseases (CVDs). A growing body of evidence suggests that metabolic disorders, characterized by AT inflammation and accumulation around organs may eventually induce organ dysfunction through a direct local mechanism. Interestingly, perirenal adipose tissue (PRAT), surrounding the kidney, influences renal function and metabolism. In this regard, PRAT emerged as an independent risk factor for chronic kidney disease (CKD) and is even correlated with CVD. Here, we review the available evidence on the impact of PRAT alteration in different metabolic states on the renal and cardiovascular function. We present a broad overview of novel insights linking cardiovascular derangements and CKD with a focus on metabolic disorders affecting PRAT. We also argue that the confluence among these pathways may open several perspectives for future pharmacological therapies against CKD and CVD possibly by modulating PRAT immunometabolism.

## Introduction

Adipose tissue (AT) is an active cellular complex that includes three different cellular types: white, brown, and beige adipocytes where extensive molecular, physiological and metabolic heterogeneity among different adipose depots exists (1–3). The main characteristic of white adipose tissue (WAT) is the large unilocular lipid droplet occupying most of the adipocyte volume. WAT functions as an excess lipid store in the form of triglycerides and secretes free fatty acids (FFA) to fulfill metabolic demands. Importantly, WAT regulates metabolic homeostasis by the synthesis and secretion of adipokines (4). Brown adipose tissue (BAT) is characterized by dispersed pockets of multilocular adipocytes and is rich in mitochondria. The main function of BAT is to dissipate energy through uncoupled respiration, that is mainly mediated by uncoupling protein-1 (UCP-1) (5). All of these AT depots are well vascularized, innervated by nerve structures, and contain preadipocytes, pericytes and immune cells (6). Recently, extensive research has uncovered the crucial role of AT depots. Not only does the physiological function of AT involve the maintenance of local and general homeostasis, *via* endocrine and paracrine activity, but also AT may contribute to the pathogenesis of many diseases (7–9). In this respect, the involvement of several fat depots was identified; perivascular adipose tissue (PVAT) is involved in the pathogenesis of hypertension (10) and epicardial AT is associated with atherosclerosis and coronary diseases (11).

Perirenal AT (PRAT) is yet another metabolically active AT depot. PRAT harbors an endocrine and paracrine role synthesizing and secreting adipokines pertinent to glucose and lipid homeostasis as well as inflammation (12). Interestingly, evidence shows that PRAT may influence the function and metabolism of the renal and cardiovascular system. Here, we summarize the recent findings regarding PRAT origin, structure and anatomical characteristics. We elaborate on the involvement of PRAT in different pathological conditions presenting new insights linking cardiovascular and renal diseases with a focus on metabolic disorders. An argument that the confluence exists among the pathways controlling metabolism and inflammation is made. This knowledge may represent a keystone for future novel approaches in metabolic, renal, and cardiovascular therapy and may open several perspectives in the field of PRAT immunometabolism.

## Perirenal Adipose Tissue: Anatomy, Histology, and Origins

PRAT, a fat depot in the retroperitoneal space surrounding the kidney, was previously believed to act as mechanical support to the kidneys (13). However, recent studies highlighted that not only PRAT has an essential role in regulating kidney function but is also involved with cardiovascular system control. Anatomical studies have confirmed that PRAT exhibits an extensive blood supply, lymphatic channels, and neuronal innervation (14–16). Due to its interaction with renal blood vessels and possible exertion of physical hemodynamic effect, PRAT is believed to modulate the renal context in a manner analogous to that of PVAT in controlling blood pressure (17, 18).

The arterial blood supply to PRAT is derived from branches of the left colic, lower adrenal, renal, lumbar and ovarian/testicular arteries. This generates an abundant anastomosing capillary network supplying PRAT with oxygen and nutrients (19). Thus, PRAT is very well vascularized and is richly innervated (15, 20).

Although studies on the origin of PRAT are limited, emerging transcriptomic data provide insights into the unique nature of PRAT (2). Recent observations on PRAT adipogenesis revealed that preadipocytes are negative for endothelial markers (21). Indeed, human PRAT has been demonstrated to be a hybrid visceral AT, analogous to subcutaneous AT, and distinct from other visceral depots (2). Nevertheless, PRAT exhibits age-dependent molecular and morphological alterations. In human embryos, PRAT-derived adipocyte progenitors differentiated *in vitro* exhibit similar features of BAT including PRDM16 and UCP1 expression, as well as a comparable mitochondrion copy number, gene expression patterns, and oxygen consumption rates (22). In newborns, PRAT predominantly consists of brown adipocytes with a thin layer of WAT, which exhibit an age-dependent, progressive regression, such that adult PRAT appears to be predominantly white with dispersed pockets of multilocular adipocytes (23, 24). However, recent studies have shown that adult PRAT comprises spatially-distinct populations of dormant unilocular and multilocular UCP1-expressing adipocytes (21, 24–26). While unilocular UCP1-expressing adipocytes are evenly distributed within PRAT, multilocular UCP1-expressing adipocytes are located around the adrenal gland, in areas containing a higher number of sympathetic nerve endings (21). These two types of AT are associated with preadipocytes, mesenchymal stem cells, and several inflammatory cells (12). PRAT arises as a focal point in regenerative medicine, it is considered a depot for mesenchymal stem cells which manifest the ability to differentiate into adipocyte, osteogenic, chondrogenic and epithelial lineage (27). BAT progenitor cells are present in PRAT regardless of specific location (21). About 30% of PRAT population expresses UCP-1, the majority being multilocular and about 20% of them exhibiting a unilocular phenotype (21, 25).

The variability in PRAT morphology is also gender-dependent, PRAT is much more developed in males than females (28). Computed tomography measurements of PRAT were carried out in 123 individuals where males had higher PRAT volume than females at a comparable waist circumference (28). Another study confirmed the gender variability in PRAT thickness and volume compared to waist circumference (29). Gender based discrepancies are also reflected in the histological pattern of PRAT. BAT in PRAT has higher expression levels of UCP-1 in females than males (30). In cold weather, PRAT can show higher levels of BAT (25). The increase in browning capacity after cold exposure in females can be observed as heat is rapidly dispersed throughout the body and is attributed to the abundance of renal blood supply. Moreover, stronger browning capacity in females is associated with specific characteristics of mesenchymal cells of PRAT and to a much lesser extent related to hormonal interventions (30). These findings are confirmed by a study on a murine model showing that Y-chromosome suppresses BAT UCP-1 expression (31).

When compared to other typical visceral AT, PRAT is more active in energy metabolism, synthesis, and secretion of several adipokines and inflammatory cytokines (12). PRAT manifests an immunoregulatory phenotype in response to several inflammatory cytokines as interleukin-1 beta (IL-1β), interferon (IFN), and tumor necrosis factor alpha (TNF-α) which could be targeted in anti-inflammatory therapy (27). These cytokines produced can regulate kidney function through paracrine or endocrine pathways. PRAT contributes to a decrease in kidney function in hypertensive individuals regardless of their body mass index (32). Furthermore, PRAT increases in prediabetic and diabetic patients and is associated with lower glomerular filtration rates in diabetic individuals (17, 18). This represents a potential immunomodulatory mechanism that could be targeted in different aspects of inflammatory conditions, tissue injuries (27), CVD and renal dysfunction.

## Perirenal Adipose Tissue Physiology

### Sympathetic Innervation

The autonomic nervous system is a key regulator of cardiovascular as well as energy homeostasis (33–35). The sympathovagal balance is essential in maintaining proper regulation of the cardiovascular and metabolic activity. Studies in human and experimental models indicate that sympathetic overflow induces hypertension (36, 37) and targeted end-organ damage (38, 39). Sympathetic nerve overactivity is detected in various tissues in obesity. Increased renal sympathetic nerve activity is reported in obese individuals and can be assessed by kidney norepinephrine spillover (40). Moreover, the sympathetic innervation regulates thermogenesis and energy liberation by innervating both the brown and white adipose pools (41–43).

**Figure 1:**
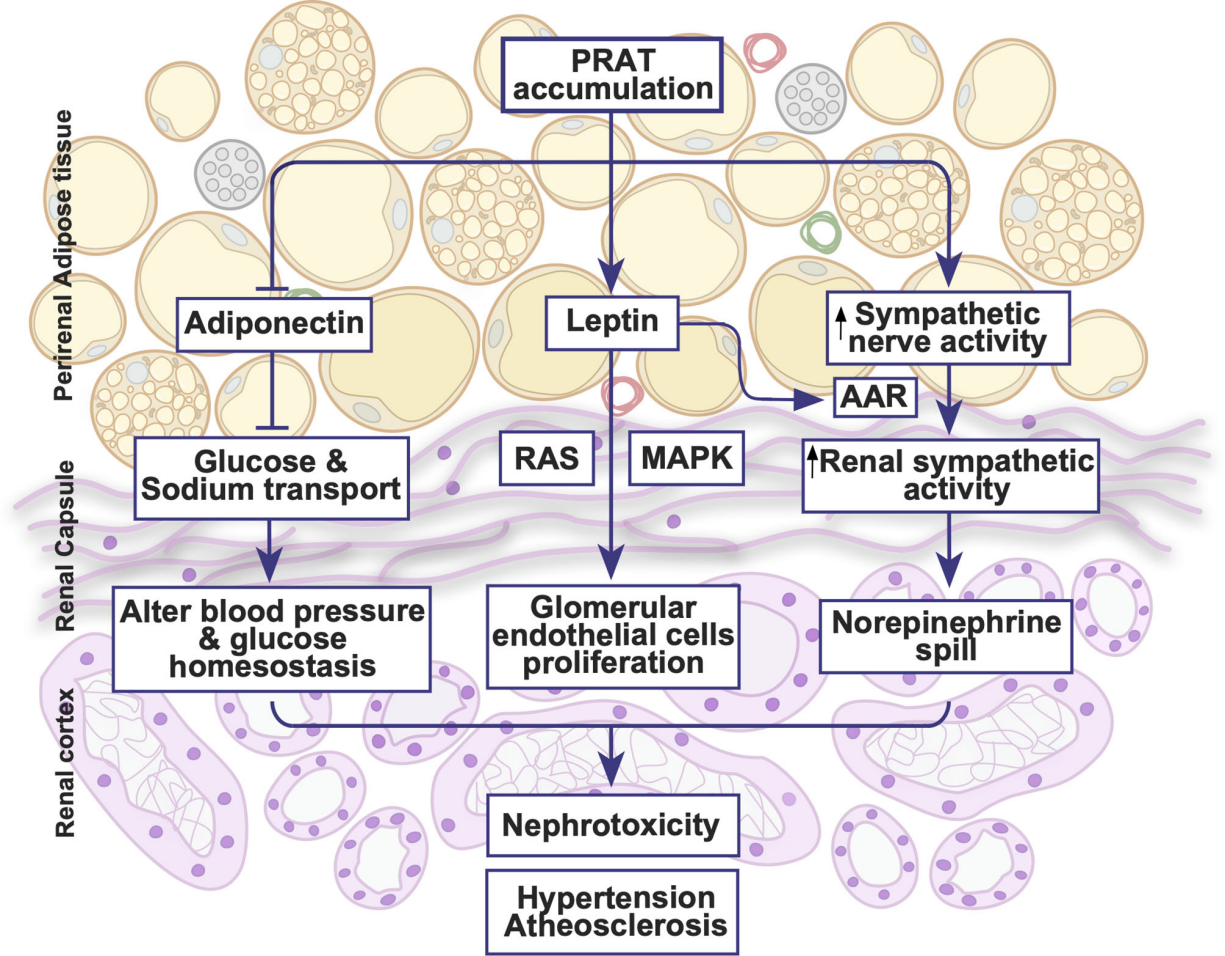
Illustrative summary of perirenal adipose tissue physiology. PRAT exhibits an extensive blood supply, lymphatic channels and neuronal innervations. PRAT accumulation activates sympathetic activity, leptin secretion and renin angiotensin system all of which could lead to hypertension, atherosclerosis and nephrotoxicity. AAR, adipose afferent reflex; MAPK, mitogen-activated protein kinase; RAS, renin angiotensin system.

The autonomic innervation into PRAT is functionally active (**Figure 1**). Indeed, the activation of afferent signals in the PRAT induces an increase in renal sympathetic activity (44). The afferent nerves in the adipocytes controlling the sympathetic outflow are referred to as an adipose afferent reflex (AAR) that modulates local homeostasis regulating energy balance and lipolysis (45–47). The activation of this sympatho-excitatory reflex in PRAT, AAR, can elevate sympathetic nerve activity and blood pressure (48, 49). The effect of the sympathetic innervation was even greater in hypertensive rat models or following a high fat diet (48, 49). Additionally, PRAT through AAR could regulate the sympathetic flow and therefore the cardiovascular system (50). Nevertheless, the function of the primary afferent neurons innervating PRAT remains unclear, further studies are essential to clarify the constituents of this pathway and the possible pathogenesis involved.

Given that PRAT thermogenic activation is essentially downstream of adrenergic stimulation, the inhibition of the sympathetic nervous system in obese rats by carotid baroreceptor stimulation not only alleviated metabolic derangements and insulin resistance, but also reduced PRAT mass and adipocyte hypertrophy, among other depots, by modulating the AMPK/PPARα/γ pathway (51). This was associated with a reduction of plasma and PRAT norepinephrine levels and an augmentation of acetylcholine levels (51). Moreover, rebalancing the autonomic nervous system by irradiating carotid baroreceptors in obese rats ameliorated metabolic dysfunction (52). This was associated with a restoration of norepinephrine and acetylcholine levels in PRAT, among other adipose depots, which rectified the AMPK/PPARα/γ pathway originally altered in obese rats (52). Although both interventions altered UCP-1 expression in BAT, neither altered UCP-1 expression in PRAT (51, 52). Nevertheless, it was demonstrated that systemic catecholamine excess in paraganglioma patients enhanced the prevalence of activated brown adipocytes in PRAT (53). Additionally, it was shown that high-fat diets as well as low protein, high carbohydrate diets induced PRAT browning that is associated with an upregulation of UCP-1, PRDM16, as well as β-AR expression (9, 54). Moreover, β_3_-AR activation in HFD-fed obese rats not only enhanced FFA influx into PRAT, but also its utilization, observations similar to those in rats fed a low protein, high carbohydrate diet (54, 55). As PRAT represents a heterogenous and unique adipose depot, contrasting data emanating from these studies must be interpreted cautiously, and depending on the context. Nevertheless, the function of the primary afferent neurons innervating PRAT remains unclear, further studies are essential to clarify the constituents of this pathway and the possible pathogenesis involved.

### Humoral Regulation-Adipokines

Adipokines encompass a group of endocrine proteins synthesized and released by adipose tissues functioning as regulators of the immune system and metabolism including insulin sensitivity and energy balance (56). These properties of adipokines are linked to metabolic dysfunction, CVD, and type 2 diabetes mellitus pathogenesis (57, 58). PRAT is highly active in adipokine synthesis as well as several pro-inflammatory cytokines (12, 59). PRAT secreted adipokines could affect the function of adjacent organs including the kidneys. Moreover, adipokines released into the systemic circulation could regulate CVS function (60, 61).

Leptin, an adipocyte-derived hormone, is a major regulator of hunger, energy homeostasis, and endocrine function (62). Circulating leptin increases in obesity through Janus kinase signal transducer and activator of transcription (JAK-STAT) pathway (63). Hyperleptinemia exacerbates atrial fibrosis and atrial fibrillation, as well as impaired glucose intolerance in obese mice (64, 65). Moreover, hyperleptinemia is associated with hypertension in obese men (66). Leptin injection into PRAT in rats stimulated the AAR without affecting the systemic sympathetic activity, highlighting that PRAT could directly regulate cardiovascular function (50). Moreover, in a rat model of metabolic syndrome (MetS), PRAT-derived leptin exacerbates the proliferation of glomerular endothelial cells by activating the MAPK pathway (67). Increased leptin synthesis in PRAT induced higher leptin concentrations in kidneys increasing the proliferation of glomerular endothelial cells through a cross-talk between the renin-angiotensin system (RAS) and leptin pathway, an effect that was reversed following the blockade of either RAS or leptin pathways (67). Therefore, aside from its systemic role, PRAT-derived leptin could directly affect endothelial cells and regulate RAS thereby affecting blood pressure as well.

Adiponectin is the most abundant adipokine in the human serum showing unique insulin-sensitizing, anti-inflammatory, cardioprotective, and antiapoptotic actions (60, 68). Adiponectin modulates metabolism; low levels of circulating adiponectin are linked to type 2 diabetes, atherosclerosis, and CVDs (69–71). Moreover, the activation of peroxisome proliferator-activated receptor delta (PPARδ) increases adiponectin secretion from PRAT, which exerts a protective effect on the renal tubular epithelial cells (72). The high salt diet-induced PPARδ activity inhibits sodium-glucose cotrasporter-2 (SGLT2) which promotes natriuresis and glycosuria. In diabetic states, patients show reduced natriuresis mainly due to impaired SGLT2 function. Moreover, reduced natriuresis in patients with uncontrolled hyperglycemia is correlated with low adiponectin levels. As a result, a distinctive role of adiponectin is revealed in regulating sodium and glucose homeostasis *via* SGLT2 in kidney tubules, a mechanism that is found to be impaired in diabetes (72).

### Renin-Angiotensin System

The renin-angiotensin system (RAS) is a crucial regulator of energy metabolism, having a major role in several metabolic disorders including obesity and insulin resistance (73). RAS modulates adipocyte function, glucose, and triglyceride metabolism as well as lipolysis (74, 75). The expression of all components of the RAS, including angiotensin II (Ang II) and angiotensin 1-7 (Ang 1-7) and their receptors, have been recognized in adipocytes implying the involvement of local RAS in regulating AT function (74, 76). Recent studies have revealed the counteractive role of Ang II and Ang1-7 in regulating various functions of adipocytes (77).

Divergent findings have been reported assessing the regulation of RAS in AT depending on the type of adipose pool and different models studied [reviewed in (78)]. In rodents, the local angiotensinogen synthesis in AT is increased following increases in food intake. In this model, angiotensinogen induced local Ang II synthesis, promoting AT growth (79). Moreover, in mice, the accumulation of AT is correlated with higher blood pressure an effect that is thought to be mediated *via* an increase in Ang II secretion from AT (80). In humans, Ang II has antiadipogenic effects in preadipocytes (81, 82). On the other hand, in obese hypertensive individuals, Ang II is increased highlighting a link between RAS and insulin resistance (83). In line, other clinical studies reported an increase in RAS components in obese individuals (84–87). Indeed, the local genetic expression of RAS in adipocytes of obese individuals is elevated (78, 88–91).

Recent studies showed that during adipogenesis, both Ang II production and Ang II type 1 receptor (AT_1_R) are upregulated (92). The stimulation of AT_1_R promotes leptin secretion in human adipocytes, an effect mediated *via* extracellular-signal-regulated kinases 1 and 2-dependent (Erk1/2) pathway (93). The stimulation of AT_1_R in AT induces the production of several pro-inflammatory cytokines (94), which in turn stimulate the apoptosis of BAT and inhibit the browning of WAT (95). However, the blockade of AT1R curbs lipid accumulation and reactive oxygen species (ROS) generation in adipocytes. This was associated with increases in adiponectin and apelin and a decrease in the TNF-α, renin, and visfatin (92, 96).

On the other hand, Ang 1-7 pathway opposes the Ang II-AT_1_R signaling, as it stimulates lipolysis and glucose uptake in the adipose pools and suppresses oxidative stress (97, 98). The activation of angiotensin-converting enzyme 2 (ACE2) *in vivo* reduces AT deposition (99). Ang 1-7 administration to rats on a high-fat diet was found to increase ACE2 expression and reduce AT accumulation (100). More studies on rats verified the previous results, in which Ang1-7 lipolytic effects were reduced *via* blockade of Mas receptors with a PI3K inhibitor (97). Nevertheless, blockers of AT_1_R and AT_2_R *in vitro* did not provoke changes in the Ang1-7 function.

PRAT fat deposition is presumed to activate RAS through compression of blood vessels, lymphatic system, and ureters, which leads to the development of hypertension, atherosclerosis and kidney dysfunction (101, 102). Low concentrations of Ang II increase adipocyte differentiation of human preadipocytes isolated from PRAT (103). Moreover, in states of metabolic dysfunction inflammatory cytokines derived from PRAT could be involved in nephrotoxicity (9, 104). The increase in PRAT inflammation in diabetic mice was reduced following Ang1-7 treatment. Additionally, Ang1-7 counteracted ROS production in PRAT (104). The functional significance of PRAT production of RAS components is an area of intense investigation, it could reveal a link between metabolic dysfunction, CVD, and CKD.

## Perirenal Adipose Tissue Inflammation and Metabolic Complications

### Mechanisms Governing Adipose Tissue Inflammation and Thermogenesis

Metabolic homeostasis is governed by balanced, intricate, and opposing processes promoting energy acquisition and energy expenditure in order to maintain basal metabolic rates (105). An imbalance of such processes, exemplified by an increased energy acquisition due to caloric excess, is thought to drive early metabolic dysfunction, eventually culminating in the emergence of insulin resistance and its accompanying derangements such as obesity, type 2 diabetes, and CVD (106). Indeed, excessive caloric intake induces hyperinsulinemia, which drives adipocyte hypertrophy, promoting the diametric expansion of the AT beyond the diffusion potential of oxygen (107–109). WAT exhibits a decreased blood supply during hypertrophic remodeling resulting in a local hypoxic state. This is accompanied by an increased adipocyte oxygen consumption that is not made up for by proper compensatory vascularization, which triggers hypoxia-inducible factor-1 alpha (HIF-1α) expression and causes adipocyte death and subsequent inflammation (108). Indeed, hypoxia induces the release of proinflammatory cytokines, chemokines and angiogenic and fibrotic factors from adipocytes, favoring AT dysfunction and immune cell infiltration (61). Hypoxia-triggered expression of HIF-1α induces NF-κB-mediated cytokine production including IL-1β, which signals for the recruitment of circulating immune cells, causing an imbalance between homeostatic AT-resident immune cells and infiltrating proinflammatory immune cells (61, 110). Therefore, obesity is considered a state of chronic low-grade inflammation, in which infiltrating immune cells contribute to the hypoxic phenotype and to insulin resistance (111, 112). Additionally, hypoxia-induced AT dysfunction is associated with an extensive lipolytic activity and free fatty acids (FFA) release, promoting endoplasmic reticulum stress and adipocyte apoptosis (113, 114). In response to adipocyte death, the AT initiates a self-limiting reparative response by which infiltrating macrophages encircle apoptotic adipocytes creating histologically-distinguishable crown-like structures (61, 115). These macrophages aberrantly generate toxic ROS and nitric oxide (NO), which further damage neighboring cells, promoting tissue fibrosis (61). As the injurious signal persists, the chronic stimulation of myofibroblasts and immune cells exacerbates tissue damage, eventually leading to extracellular matrix remodeling, tissue fibrosis, and AT dysfunction (116).

There exists extensive heterogeneity among different adipose depots and among the adipocytes of a given depot itself, resulting in differential, depot-specific susceptibilities to inflammation (1–3). A long standing subcategorization of adipose depots differentiates between WAT and BAT. WAT comprises unilocular adipocytes that specialize in energy storage and adipokine secretion while BAT comprises mitochondria-rich, multilocular adipocytes that specialize in energy dissipation through non-shivering thermogenesis (5). Non-shivering thermogenesis encompasses intricate thermogenic pathways that are thought to occur downstream of β_3_-adrenergic receptors (β_3_-ARs), and in response to stimuli that enhance local sympathetic discharge including cold exposure and high fat diet consumption (5, 117, 118). These latter stimuli promote WAT browning, a phenomenon by which white adipocytes gain thermogenic potential, transforming into brown-like beige adipocytes. Emerging evidence implicates different thermogenic pathways downstream of β_3_-ARs, that drive the thermogenic potential of brown and beige adipocytes. The most efficient and quantitatively significant thermogenic effector is the inner mitochondrial membrane protein, UCP-1. UCP-1 is a fatty acid/H^+^ symporter that uncouples mitochondrial oxidative phosphorylation from the production of ATP (5). Moreover, the activation of β_3_-ARs induces lipolysis, increasing the levels of FFA, which further enhances UCP-1 activity. Nevertheless, it was shown that UCP-1 is dispensable for cold-induced and diet-induced thermogenesis (119). It was therefore hypothesized that less-efficient thermogenic pathways contribute to adaptive thermogenesis, the most prominent of which is creatine futile cycling (120). Creatine futile cycling, that is the phosphorylation of creatine by creatine kinase B and its subsequent futile hydrolysis, appears to take place in UCP-1-positive and UCP1-negative adipocytes (121, 122). Importantly, blocking creatine cycling in adipocytes either by impairing its endogenous biosynthesis or its transport, promotes diet-induced obesity and cold-intolerance in mice (123–125). Alternative thermogenic pathways also include lipolysis/re-esterification cycling that mediates adaptive thermogenesis based on the ATP demand of triacylglycerol synthesis, calcium cycling that is mediated by the SR/ER calcium ATPase pump and phospholamban, and the UCP1-independent proton leak by the mitochondrial ADP/ATP carrier that is initiated at high membrane potentials (5). Importantly, UCP1-dependent and UCP1-independent uncoupling of mitochondrial respiration from ATP production is linked to aberrant increases in the mitochondrial oxygen consumption rates and oxygen demand, thus possibly exacerbating AT hypoxia in states of metabolic dysfunction (7–9, 126–128).

### Mechanisms Linking Perirenal Adipose Tissue Thermogenesis and Inflammation

Several studies have shown that an augmentation of central adiposity in overweight and obese individuals is associated with an increased PRAT mass, and that PRAT mass independently associates with insulin resistance and lower HDL-cholesterol levels (129). Moreover, PRAT thickness is associated with cardiovascular risk factors in a sex-dependent manner, where significant associations exist between increased PRAT thickness and fasting plasma glucose level, metabolic syndrome, and waist circumference in men, and only fasting plasma glucose level in women (130). Nevertheless, such observations cannot be merely explained by an increased PRAT mass. Recent mechanistic investigations possibly link PRAT thermogenesis and inflammation to renal dysfunction early in the course of metabolic disease (9). Although it could be inferred from clinical observations linking reduced PRAT browning to hypertension and metabolic dysfunction that PRAT browning holds therapeutic benefits, recent evidence in non-obese, prediabetic rats, links PRAT thermogenesis to localized inflammation, impairing renovascular function early in the course of metabolic disease (9, 131).

Indeed, increased UCP1 expression is consistently reported in different animal models of diet-induced obesity, a phenotype that is enhanced by the increased abundance of long chain fatty acids pertinent to these models (132, 133). Such an increased expression level of UCP1 drives diet-induced thermogenesis and is associated with increased levels of oxygen consumption (134). This is of particular relevance to adipose depots with inherently low expression levels of UCP1, such as the PRAT, in which diet-induced thermogenesis produces profound bioenergetic and inflammatory alterations (9). Indeed, PRAT inflammation and enhanced oxidative stress in prediabetic rats were associated with elevated glomerular filtration rate (GFR) accompanied by mild proteinuria in the absence of hypertension, hyperglycemia, obesity, and systemic inflammation (9). These rats exhibited an acutely increased production of ROS in PRAT which is suggested to have enhanced UCP-1 activity and mitochondrial respiration uncoupling (9, 135). Importantly, PRAT of HFD-fed rats exhibited an enhanced expression of UCP-1 which is thought to exacerbate local hypoxia and increase HIF-1α expression in the hypertrophied tissue by driving the aberrant consumption of oxygen. While studies of the PRAT UCP1 expression profile are scarce, the available evidence shows alteration of UCP1 expression in PRAT adipocytes in disease conditions such as hypertension and renal cell carcinoma (131, 136). In this context, mechanistic parallels can be drawn to PVAT, an intrinsically hybrid tissue harboring brown adipocytes, in which an increased UCP-1 expression exacerbates local hypoxia leading to AT dysfunction and inflammation and subsequent vascular derangements (8, 137). It was therefore suggested that UCP-1 may serve as a therapeutic target in select adipose depots to mitigate cardiovascular and renovascular derangements associated with early phases of metabolic dysfunction (61, 137). This is of particular relevance as the upregulation of PRAT UCP-1 expression and the excessive uncoupling of mitochondrial respiration not only deteriorated kidney function but were also associated with altered expression of epithelial and mesenchymal markers supportive of renal epithelial to mesenchymal transition (9).

Additionally, it was shown that PRAT altered adipokine profile and enhanced oxidative stress, inflammation, and fibrosis may partly explain the high risk of cardiovascular events observed in patients with primary aldosteronism or hypercortisolism (138, 139). In patients with aldosterone-producing and cortisol-producing adenomas, PRAT expressed significantly higher levels of IL-6 and TNF-α as well as fibrotic markers in comparison to normotensive individuals and patients with essential hypertension (138, 139). These observations are supported by *in vitro* experiments showing that aldosterone treatment of isolated human PRAT stromovascular cells, mouse 3T3-L1, and brown preadipocytes induces the expression of IL-6 and markers of inflammation and fibrosis (138). Additionally, the expression level of NADPH oxidase 4 (NOX4) significantly increased, while that of hemoxygenase-1 (HO-1) and nuclear factor erythroid 2-related factor 2 (Nrf2) significantly decreased in PRAT of patients with cortisol-producing adenoma (139). Indeed, dexamethasone treatment of pre-differentiated stromovascular cells, mouse 3T3-L1, and brown preadipocytes induces marked fibrosis and adipogenesis (139). Moreover, it was shown that dexamethasone treatment in adrenalectomized rats promotes hyperplastic PRAT expansion that is associated with an increased expression and dehydrogenase activity of 11 β-hydroxysteroid dehydrogenase type 1, an NADPH-dependent cortisone reductase (140). Moreover, hypercortisolism in patients with active Cushing’s syndrome induced PRAT adipocyte hypertrophy, that was associated with an increased macrophage infiltration, and augmented leptin and reduced adiponectin levels (141). Additionally, PRAT brown adipocytes were shown to be active in states of secondary hyperaldosteronism, such as in patients with pheochromocytoma (142). Nevertheless, the inflammatory landscape of PRAT in these patients was not assessed. However, it could be inferred from these studies that states of hyperaldosteronism and hypercortisolism drive both PRAT inflammation and increased UCP-1 indicating that possible cross-talks between both mechanisms plausibly exist.

## Perirenal Adipose Tissue and Renal Diseases

### PRAT and Chronic Kidney Disease: A Possible Correlation

The strong correlation between body mass index and higher risk of chronic kidney disease (CKD) was first reported in 1974 and was found to be greatly associated with an increase in proteinuria (143). Patients with a high body mass index had a higher risk of CKD compared to lean individuals independent of age (144). Moreover, urinary albumin excretion was also elevated in obese patients who are neither hypertensive nor diabetic (145). Indeed, CKD is an independent complication of obesity; a metabolic profile identified as obesity-related glomerulopathy or obesity-related kidney disease (146, 147). Several studies highlighted the association between increased visceral adiposity, and particularly increased PRAT mass and volume, and determinants of metabolic and CV disease as well as kidney dysfunction (129, 148). Excess PRAT has also been associated with metabolic anomalies including insulin resistance, abnormal fasting blood glucose levels, hypertriglycemia, and higher uric acid excretion in urine in patients with CKD (129, 148).

Given the intimate relation between PRAT and the kidney, either due to their spatial proximity or due to their common innervation and vascularization, PRAT expansion, dysfunction, and inflammation are thought to have a pronounced impact on renal function (149, 150). PRAT thickness is increased in patients with MetS, which is accompanied by increases in oxidative stress and renal microvascular proliferation (151). PRAT expansion secondary to obesity contributes to kidney dysfunction irrespective of obesity (12, 32). This is in line with the observation that abdominal obesity was strongly associated with CKD compared to overall obesity (152, 153), where an increasing body of evidence has suggested that PRAT thickness is positively associated with visceral adiposity and waist circumference (145, 154). In this regard, excess PRAT was associated with a higher risk for CKD and could be used as a predictor for reduced GFR (145, 155, 156) and higher incidence of proteinuria in obese/overweight individuals (144, 157). A positive correlation was consistently found between PRAT thickness and microalbuminuria in obese patients (145, 156). Importantly, excessive PRAT inflammation is believed to exacerbate renal vascular and endothelial damage (12, 59, 158, 159). Moreover, it was recently shown that PRAT exhibits an age-dependent inflammatory signature that is characterized by an increased peri-organ recruitment of macrophages and inflammatory natural killer (NK) cells in the vascular stromal fraction that was associated with a deleterious impact on the microenvironment of renal transplants (160, 161). Nevertheless, another study failed to arrive at such association, where PRAT inflammation did not correlate with the reduced early renal graft function observed in obese kidney donors (162). Based on the previous findings, excess PRAT could be considered an independent predictor of CKD. Additionally, the ultrasound evaluation of PRAT is now proposed as a parameter that might be useful for early assessment of obesity-induced renal dysfunction (145).

### Possible Mechanisms

**Figure 2:**
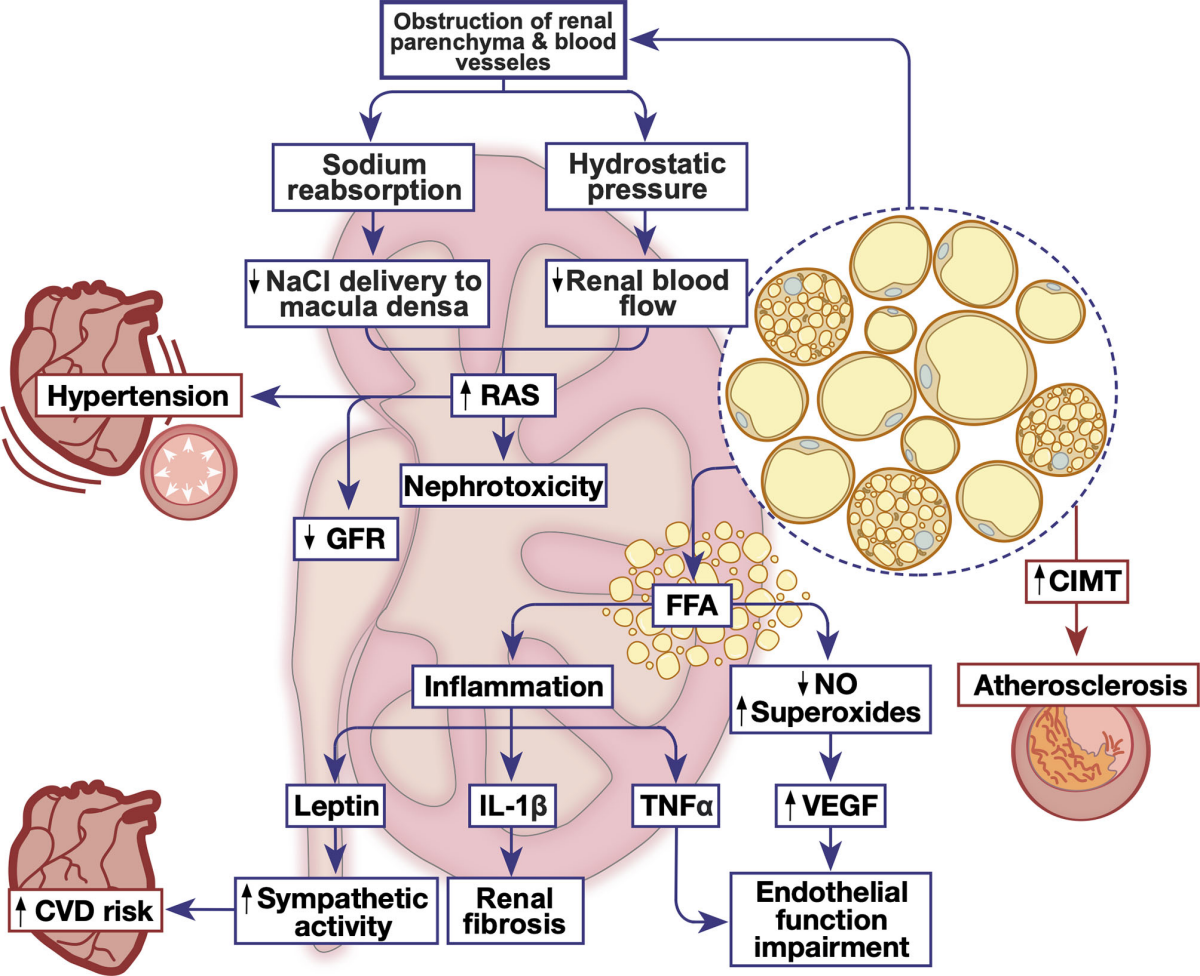
The emerging role of PRAT in renal and cardiovascular homeostatic function. Metabolic dysfunction triggers PRAT deposition and inflammation leading to alterations in cardiovascular and renal function, triggering nephrotoxicity and cardiovascular diseases. CIMT, carotid intima-media thickness; CVD, cardiovascular disease; FFA, free fatty acid; GFR, glomerular filtration rate; IL-1β, interleukin-1 beta; NO, nitric oxide; RAS, renin angiotensin system; TNFα, tumor necrosis factor alpha; VEGF, vascular endothelial growth factor.

The detailed mechanisms by which PRAT initiates and exacerbates chronic renal injury have not been completely elucidated, but several pathways are postulated (**Figure 2** and **Table 1**). First, the accumulation of PRAT on the kidney may result in a direct obstruction of renal parenchyma and vessels. This might induce increases in sodium reabsorption and higher blood pressure as a consequence in addition to alterations in kidney function in obese patients (24). The firm encapsulation of the kidney could further increase the interstitial hydrostatic pressure and reduce renal blood flow (149, 167). Consequently, the increase is sodium absorption leads to a decreased sodium chloride delivery to the macula densa resulting in lower resistance in afferent arterioles which leads to increased GFR and altered RAS (167–170). All of these processes including an increase in interstitial hydrostatic pressure, stimulation of renin release, glomerular filtration and sodium tubular reabsorption accelerate renal disease progression eventually leading to GFR reduction (24, 148, 149). Recently, a study on hypertensive individuals has reported a decline in GFR that was correlated to an increase in PRAT specifically among other visceral AT depot regardless of gender (32). As a consequence of GFR reduction, high uric acid and triglycerides were correlated with PRAT thickness in patients with CKD (24, 148).

**Table 1:** Main findings of studies linking perirenal adipose tissue to renal disorders.

Main finding	Possible mechanism	Conducted on	Reference
PRAT is associated with increased urinary albumin excretion	Low adiponectin and elevated leptin levels trigger pathways augmenting renal inflammatory and oxidative stress leading to renal vascular dysfunction causing increased urinary albumin excretion	obese rats	(163)
PRAT promotes renal arterial endothelial dysfunction	Accumulation of PRAT showed increases in inflammation and oxidative stress, which triggered renal endothelial dysfunction *via* TNF-α acting in a paracrine manner.	Pigs with obesity and metabolic derangements	(158)
PRAT accumulation was correlated with a decline in GFR	–	Hypertensive patients	(32)
PRAT thickness was negatively correlated with GFR	–	Diabetic patients	(164)
PRAT-derived leptin has a detrimental effect on the kidney	PRAT hypertrophy induces an increase in leptin expression that is accompanied by an imbalance in the expression of the Ang II–AT_1_R and ACE2–Ang(1–7)–Mas receptor axes. This promotes glomerular endothelial cells proliferation by activating p38 MAPK pathway.	Rats with metabolic syndrome	(67)
PRAT inflammation and macrophage infiltration are linked to high fat diet induced nephropathy	Expression of plasminogen activator inhibitor-1 (PAI-1) in PRAT was increased. PAI-1 contributes to macrophage mediated inflammation, extracellular matrix accumulation and thus diabetic nephropathy.	Obese mouse model	(165)
Localized PRAT inflammation evoked renal impairment in early course of metabolic deterioration	The paracrine effects of PRAT inflammation, presented as higher IL-1β expression, lead to renovascular endothelial dysfunction, hyperfiltration, renal cortical inflammation and proteinuria.	Non-obese prediabetic rats	(9)
PRAT Inflammation exacerbates diabetic nephropathy.	Ang1-7 supplementation to these mice not only reduced renal mesangial expansion and urinary albumin secretion, but also ameliorated renal fibrosis and PRAT oxidative stress and inflammation mainly through the attenuation of NOX-mediated ROS production.	*db/db* mice	(166)
Excessive perirenal adiposity may constitute an independent prognostic factor of kidney malfunction	Inducing the heme oxygenase system in diabetic fat rats reduced PRAT adiposity, macrophage infiltration, and the production of pro-inflammatory cytokines such as TNF-α, IL-1β, and IL-6.	Zucker-diabetic fatty rats	(159)

Second, chronic inflammation due to the increase in FFA production is the hallmark of obesity and therefore can also be associated with modulation of PRAT function. Indeed, the increased volume of PRAT is positively associated with overproduction of FFA (145). Metabolites of FFA have a direct renal lipotoxic effect and are directly correlated with albuminuria (145, 171). Levels of FFA in renal venous blood were significantly higher than those in the jugular vein, this indicates the involvement of PRAT in kidney damage through direct intercellular signaling pathways (163). The excessive release of FFA by PRAT could directly impair endothelial function by enhancing the oxidation of tetrahydrobiopterin and uncoupling of endothelial nitric oxide (NO) synthase, leading to the production of L-arginine or superoxide instead of NO (172). The reduced NO synthesis could lead to a compensatory mechanism synthesizing vascular endothelial growth factor, leading to a greater albumin leak from the glomerulus (172). Furthermore, FFA-induced renal lipotoxicity could exacerbate chronic inflammation by increasing the metabolism of intracellular fatty acids (171).

Third, excess PRAT can affect renal function through a local or systemic secretion of pro-inflammatory mediators which may influence the kidney function in a paracrine manner (12, 21, 170, 173). In obese swine, excess PRAT secretes tumor necrosis factor-α (TNF- α) which impairs renal endothelial function (158). This could be also related to the loss of NO as discussed previously. Moreover, the expression of plasminogen activator inhibitor-1 (PAI-1) in PRAT was increased in high-fat diet-induced obese mouse model (165). PAI-1 is shown to contribute to macrophage mediated inflammation, extracellular matrix accumulation and thus diabetic nephropathy. The genetic deletion or the inhibition of PAI-1 reduced PRAT inflammation, renal histological lesions and abated renal fibrosis (165). Excessive PRAT secretion of TNF- α and leptin could trigger renal fibrosis in murine models (29, 174, 175). Leptin exacerbates vascular remodeling and glomerular proliferation *via* activation of the p38 MAPK pathway (67). Concomitantly, TNF- α induces direct renal endothelial dysfunction thus modulating GFR (67, 158). Leptin secretion could also stimulate the sympathetic nervous activity *via* altering the proopiomelanocortin-melanocortin 4 receptor pathway in the central nervous system (160). Interestingly, our recent study on non-obese prediabetic rats has shown that a localized PRAT inflammation, presenting as higher IL-1β expression, evoked renal structural and functional deterioration associated with an altered renovascular endothelial function (9). Importantly, a recent study reported a direct correlation between age and inflammatory profile of donor-derived stromal vascular fraction of PRAT (PRAT-SVF), expressed by a local recruitment of NK cells. These NK cells are associated with NKG2D receptor activation and encodes for INFγ, indicating that NK cells derived could be involved in the pro-inflammatory pathway leading to renal dysfunction in elderly patients with transplants (160).

Additionally, as both obesity, diabetic nephropathy, and primary hypertension are associated with an enhanced activity of the RAS, it becomes plausible that the enhanced activity of this system in PRAT and in the adrenal gland participates in the pathogenesis of obesity-associated hypertension and diabetic nephropathy, particularly through Ang II-mediated PRAT dysfunction (101, 102). The inhibition of ACE in a uninephrectomized rat model of chronic renal dysfunction prevented PRAT mass accumulation and led to the emergence of multilocular thermogenic adipocytes (176). Indeed, it was shown that Ang1-7 counteracts the detrimental effects of Ang II on diabetic nephropathy in db/db mice (166). Ang1-7 supplementation to these mice not only reduced renal mesangial expansion and urinary albumin secretion, but also renal fibrosis and PRAT oxidative stress and inflammation mainly through the attenuation of NOX-mediated ROS production (166). Nevertheless, it remains unclear whether Ang1-7 reduced renal dysfunction by virtue of its ameliorative effect on PRAT inflammatory and oxidative status. Moreover, such mechanisms contributing to diabetic nephropathy were shown in Zucker-diabetic fatty rats (159). Inducing the heme oxygenase system in these rats reduced PRAT adiposity, macrophage infiltration, and the production of pro-inflammatory cytokines such as TNF-α, IL-1β, and IL-6 (159). Moreover, the hemin-mediated enhancement of the heme oxygenase system in PRAT reduced the proinflammatory M1 macrophage polarization while inducing the anti-inflammatory M2 macrophage polarization. These effects were accompanied by reduced renal damage and fibrosis and enhanced kidney function. As these models exhibit extensive damage that is associated with diabetes, the dissection of a causality relationship between PRAT inflammation and renal dysfunction remains marginally possible. Another study in non-diabetic, high fat diet-fed rats highlighted PRAT association with increased urinary albumin excretion due to renal vascular dysfunction caused by the activation of renal inflammatory and oxidative stress augmenting pathways (163). Nevertheless, the inflammatory condition of PRAT was not assessed and thus these observations were attributed to increased visceral adiposity, and particularly increased PRAT mass. However, it was shown in non-obese prediabetic rats that subtle PRAT inflammation induces pronounced kidney dysfunction in isolation of hyperglycemia and systemic inflammation (9). It therefore becomes plausible that PRAT inflammation in the setting of metabolic dysfunction might precede renal manifestations of the disease.

PRAT thickness could aggravate renal anomalies due to metabolic dysfunction such as abnormal insulin serum levels, high glucose or triglycerides and uric acid, all of which are reported in patients with CKD (129). Additionally, excess PRAT is detected in patients with calcium phosphate apatite or uric acid nephrolithiasis (177). However, future research into analyzing the detailed mechanism is warranted.

## Renal Diseases and Cardiovascular Dysfunction: Cardiorenal Syndrome

A strong relationship exists between the heart and kidney where the proper function of one depends on the other. Cardiorenal syndrome is defined as ‘disorders of the heart and kidney whereby acute or chronic dysfunction in one organ may induce acute or chronic dysfunction of the other’ ultimately leading to failure of both organs (178). CKD is strongly associated with CVD, it has been reported that patients with advanced kidney disease are at high risk of CVD mortality and morbidity (179). Recent studies reported a 40-50% cardiovascular mortality rate in patients with advanced and end-stage CKD compared to 26% in patients with normal kidney function (180, 181). Nonetheless, the correction of the classical cardiovascular risk factors, including hypertension, diabetes, and dyslipidemia, in patients with CKD was not sufficient in neutralizing the impact of CKD on cardiovascular risk (182). It is also now being recognized that patients with early stages of CKD also suffer from a high risk of cardiovascular events (183) possibly implicating a role for PRAT inflammation in this observations. Consistent with these observations, CKD is now considered an independent risk factor for CVD (184). It is also worth mentioning that patients with CKD are more likely to die from CVD rather than terminal end-stage kidney disease (185).

## Perirenal Adipose Tissue and Cardiovascular Dysfunction

Obesity and specifically visceral fat accumulation are actively involved in the pathogenesis of CVDs including hypertension (186) and coronary heart diseases (CHD) (187). Abdominal obesity has a close association with MetS and CVD (188). Excess visceral AT poses a high risk for dyslipidemia, hypertension and cardiorenal disorders (189). Studies have shown that risk of CVDs is more closely related to visceral fat volume, including PRAT, compared to subcutaneous AT (130, 148, 190). However, surgical lipectomy of abdominal AT depots was not effective in improving metabolic function or reducing CVD risk (191). Therefore, a need exists to identify other AT pools in which certain interventions could lower the risk of developing CVDs. Notably, recent findings have identified PRAT specifically as an emerging risk for CVD independent of metabolic profile (190) (**Figure 2** and **Table 2**).

**Table 2:** Main findings of studies linking perirenal adipose tissue to cardiovascular disorders.

Main finding	Targeted population and references
PRAT accumulation was correlated with higher blood pressure, which was also dependent on urinary concentrations of aldosterone independent of metabolic parameters.	Obese and overweight individuals (190)
A significant direct correlation between PRAT thickness and hypertension	Hypertensive and non-hypertensive obese individuals (192)
	Patients with polycystic ovary syndrome (193)
PRAT thickness has shown to be significantly correlated with indices that predict atherosclerosis.	Male and female subjects (194)
PRAT thickness was associated with atherosclerosis specifically intima-media thickness of the common carotid artery	Male subjects (195)
	HIV-infected patients (196)
PRAT was associated with diverse metabolic and cardiovascular risk factors including carotid intima-media thickness	Asymptomatic prepubertal Caucasian children (197)

Hypertension is a common manifestation of CVD and is even associated with a higher risk of all-cause mortality (198). Hypertension increases the risk of myocardial infarction, congestive heart diseases, and stroke (12). Proper control of blood pressure is fundamental in CVD treatment and prevention of further complications. There is a linear relationship between hypertension and obesity, around 60-76% of obese individuals have hypertension (199). Indeed, PRAT thickness is positively correlated with blood pressure in obese and overweight patients (190). In obese individuals, PRAT thickness is considered as an integral parameter defining both the risk for developing arterial hypertension and chronic renal disease (192). Twenty-four hours mean diastolic blood pressure was reported to be a dependent variable of PRAT and aldosterone production independent of metabolic parameters (190). Similar results were reported in patients with polycystic ovary syndrome, a positive correlation between PRAT thickness and blood pressure in patients was documented (193). Excessive PRAT accumulation compresses the kidney, increase RAS sodium reabsorption, and induce hypertension (149, 168). There is evidence that PRAT could directly regulate the cardiovascular system through an amplified afferent signals by the AAR, resulting in an increase in the renal sympathomimetic outflow (200). PRAT hypertrophy activates macrophage infiltration and proinflammatory cytokine release that adversely affect systemic vascular function (201). Altogether, these results suggest that PRAT has a direct role in controlling blood pressure, however; further studies are recommended to assess the detailed mechanistic pathway behind this phenomenon.

Additionally, atherosclerosis is a key factor in inducing CVDs and a leading cause of morbidity and mortality (202). Carotid intima-media thickness (CIMT) assessment is considered as a marker of subclinical atherosclerosis (203) and is therefore used to determine the risk of CVDs in clinical trials (204). Indices of atherosclerosis including body weight, waist circumference can predict CHD. Alterations in AT metabolism underlie atherosclerosis and thus CHD (205). More specifically, clinical studies reported that body fat distribution has a significant correlation with the severity of CHD without any clinical evidence of CVD. Compared to other fat depots, PRAT thickness had the highest partial correlation coefficient with CVD, highlighting the contribution of PRAT in early cardiovascular changes in males and females (12). Notably, excess PRAT is positively correlated with indices that predict atherosclerosis as CIMT, waist to height and waist to hip ratio, waist circumference, and abdominal height (194). A thickness level of 0.26 cm of PRAT, measured using abdominal ultrasound, was found to determine the presence of sub-clinical atherosclerosis (194). Moreover, in HIV-infected patients, having a high risk of CVD, similar results were obtained. Patients with HIV and visceral adiposity exhibited high PRAT thickness and intima-media thickness of the common carotid artery. This implies the association of PRAT thickness and atherosclerosis in these individuals (196). Importantly, the correlation between PRAT and CIMT was even detected in children (197). Being readily available, abdominal ultrasound can easily measure PRAT thickness and therefore a prompt initiation of action to mitigate or prevent atherosclerosis and control CVD can be achieved.

Considering the above-mentioned associations, excess PRAT contributes to alterations in vascular and metabolic functions associated with CVD. PRAT is also potentially related to epicardial fat, both having mesothelial layers enriched with WAT progenitors that produce adipocytes (129). The coexistence of metabolic dysfunction, hypertension, and inflammation impacts end organ function gradually initiating a cascade of events that exacerbate CVD making it more resistant to treatment. These findings confirm the suggested hypothesis that excess PRAT is a risk factor of CVDs and a predictor for cardiac dysfunction.

## Future Perspective and Possible Treatment

Proper understanding of the molecular alterations regulating AT dysfunction could identify therapeutic targets to promote healthy AT expansion, hence preserving renal and cardiovascular function. AT dysfunction is the central mechanism for the development of complications related to obesity and metabolic diseases; therefore, most of the therapeutic approaches to mitigate AT dysfunction are related to indices of obesity (206). Interventions targeting AT dysfunction rely on a foundation of lifestyle modification including low caloric intake, suitable exercise, and fasting (207). Pharmacological tools and surgeries could be implemented to help the patients reach their health goals (207). As the potential harmful effects of excessive PRAT accumulation in renal and cardiovascular diseases were outlined, targeting PRAT inflammation emerges as a potential approach to combat the complex mechanisms implicated in CKD and CVDs.

Bariatric surgery is an effective intervention in controlling type 2 diabetes, dyslipidemia and quality of life of morbidly obese patients (208). Ricci et al. (192) showed that bariatric surgery could significantly decrease PRAT as well as blood pressure in morbidly obese patients. Moreover, as previously mentioned, PRAT is capable of reactivating dormant BAT into active BAT by cold exposure or stimulation of β_3_- adrenergic receptors. This property represents a promising strategy to combat AT inflammatory conditions leading to metabolic diseases (25, 55).

Possible pharmacological interventions include several drug classes. Beyond their lipid-lowering effects, statins, 3-hydroxy-3-methyl-glutaryl-coenzyme A (HMG-CoA) reductase inhibitors, were found to have anti-inflammatory, anti-oxidant and anti-proliferative properties (209). Statins also increase adiponectin levels, protect the endothelium and reduce urinary albumin excretion rate in patients with diabetes (210, 211). Pravastatin treatment for 6 months in patients with documented coronary artery diseases significantly increase serum adiponectin concentration (212). Higher adiponectin levels following statin treatment improves endothelial function (213), inhibit vascular smooth muscle cell proliferation (214, 215), and modulate vascular inflammatory cascades (216, 217). Despite these findings, to date no data link statin treatment with changes in the PRAT depot, however one could speculate a beneficial role of statins in PRAT dysfunction.

Other pharmacological tools to modulate PRAT inflammation are anti-diabetic drugs namely, metformin and pioglitazone. A two-week treatment with non-hypoglycemic doses of either drug was shown to ameliorate PRAT inflammation in pre-diabetic rats on mild hypercaloric diet. Interestingly, treatment reversed the deterioration of renal function triggered by 12 weeks of a mild hypercaloric feeding (9). Interestingly, a recent study showed treatment with a sodium/glucose co-transporter 2 (SGLT2) inhibitor reduced urinary albumin excretion and glomerular cell proliferation in high-fat fed mice, which was accompanied with decreased PRAT inflammation and leptin production (218). Indeed, the impact of SGLT2 inhibitors on PRAT inflammation is proposed to proceed through the activation of AMPK/Sirt1 pathway and potential restoration of autophagy (219). Moreover, short-term treatment with glucagon-like peptide receptor agonists was shown to reduce PRAT thickness and improve renovascular function in diabetic patients (220). Specifically, liraglutide treatment reduced perirenal adipocyte size in diabetic mice (221). Nevertheless, identifying specific drugs to target PRAT accumulation or inflammation require further investigation and research.

## Conclusion

This era is marked by major changes in the traditional perception of AT physiological and metabolic function. The proximity of PRAT to the kidney makes its specific anatomical and morphological features relevant to renal function and general homeostasis. In this context, regardless of the small size of PRAT compared to visceral or subcutaneous AT, the effect of the adipokines and cytokines secreted broadens the impact of PRAT in maintaining metabolic, renal, and cardiovascular homeostasis. The data concerning the unique nature and pathophysiology of PRAT is limited, but studies are underway to unveil the potential molecular factors involved in PRAT function opening promising perspectives in developing appropriate therapeutic and preventive approaches.

## Author Contributions

SH and IA participated in literature review and screening and contributed to manuscript writing. SH wrote the first draft of the manuscript. AE and YA helped inn overseeing and coordinating the work and participated in manuscript draft review. AE-Y developed the idea, supervised the work, reviewed and modified manuscript draft, and provided research funding support. All authors contributed to the article and approved the submitted version.

## Funding

This work was supported by AUB-Faculty of Medicine Medical Practice Plan Grant #320148 and an AUB President Collaborative Research Stimulus Grant to AE-Y.

## Conflict of Interest

The authors declare that the research was conducted in the absence of any commercial or financial relationships that could be construed as a potential conflict of interest.

## Publisher’s Note

All claims expressed in this article are solely those of the authors and do not necessarily represent those of their affiliated organizations, or those of the publisher, the editors and the reviewers. Any product that may be evaluated in this article, or claim that may be made by its manufacturer, is not guaranteed or endorsed by the publisher.

## References

[1] Corvera S. Cellular Heterogeneity in Adipose Tissues. Annu Rev Physiol (2021) 83:257–78. 10.1146/annurev-physiol-031620-095446 PMC8091658 33566675

[2] Schleinitz D Krause K Wohland T Gebhardt C Linder N Stumvoll M. Identification of Distinct Transcriptome Signatures of Human Adipose Tissue From Fifteen Depots. Eur J Hum Genet (2020) 28(12):1714–25. 10.1038/s41431-020-0681-1 PMC7784683 32661330

[3] Sierra Rojas JX García-San Frutos M Horrillo D Lauzurica N Oliveros E Carrascosa JM. Differential Development of Inflammation and Insulin Resistance in Different Adipose Tissue Depots Along Aging in Wistar Rats: Effects of Caloric Restriction. J Gerontol A Biol Sci Med Sci (2016) 71(3):310–22. 10.1093/gerona/glv117 26419977

[4] Giralt M Villarroya F. White, Brown, Beige/Brite: Different Adipose Cells for Different Functions? Endocrinology (2013) 154(9):2992–3000. 10.1210/en.2013-1403 23782940

[5] Chouchani ET Kazak L Spiegelman BM. New Advances in Adaptive Thermogenesis: UCP1 and Beyond. Cell Metab (2019) 29(1):27–37. 10.1016/j.cmet.2018.11.002 30503034

[6] Hildebrand S Stümer J Pfeifer A. PVAT and its Relation to Brown, Beige, and White Adipose Tissue in Development and Function. Front Physiol (2018) 9:70. 10.3389/fphys.2018.00070 29467675 PMC5808192

[7] Schaffer JE. Lipotoxicity: When Tissues Overeat. Curr Opin Lipidol (2003) 14(3):281–7. 10.1097/00041433-200306000-00008 12840659

[8] Elkhatib MAW Mroueh A Rafeh RW Sleiman F Fouad H Saad EI. Amelioration of Perivascular Adipose Inflammation Reverses Vascular Dysfunction in a Model of Nonobese Prediabetic Metabolic Challenge: Potential Role of Antidiabetic Drugs. Transl Res (2019) 214:121–43. 10.1016/j.trsl.2019.07.009 31408626

[9] Hammoud SH AlZaim I Mougharbil N Koubar S Eid AH Eid AA. Peri-Renal Adipose Inflammation Contributes to Renal Dysfunction in a non-Obese Prediabetic Rat Model: Role of Anti-Diabetic Drugs. Biochem Pharmacol (2021) 186:114491. 10.1016/j.bcp.2021.114491 33647265

[10] Stieber C Malka K Boucher JM Liaw L. Human Perivascular Adipose Tissue as a Regulator of the Vascular Microenvironment and Diseases of the Coronary Artery and Aorta. J Cardiol Cardiovasc Sci (2019) 3(4):10–5. 10.29245/2578-3025/2019/4.1174 PMC7224402 32411947

[11] Liu Z Wang S Wang Y Zhou N Shu J Stamm C. Association of Epicardial Adipose Tissue Attenuation With Coronary Atherosclerosis in Patients With a High Risk of Coronary Artery Disease. Atherosclerosis (2019) 284:230–6. 10.1016/j.atherosclerosis.2019.01.033 30777338

[12] Liu BX Sun W Kong XQ. Perirenal Fat: A Unique Fat Pad and Potential Target for Cardiovascular Disease. Angiology (2019) 70(7):584–93. 10.1177/0003319718799967 30301366

[13] Marx WJ Patel SK. Renal Fascia: Its Radiographic Importance. Urology (1979) 13(1):1–7. 10.1016/0090-4295(79)90002-5 442312

[14] Kim JH Han EH Jin ZW Lee HK Fujimiya M Murakami G. Fetal Topographical Anatomy of the Upper Abdominal Lymphatics: Its Specific Features in Comparison With Other Abdominopelvic Regions. Anat Rec (Hoboken) (2012) 295(1):91–104. 10.1002/ar.21527 22144396

[15] Czaja K Kraeling R Klimczuk M Franke-Radowiecka A Sienkiewicz W Lakomy M. Distribution of Ganglionic Sympathetic Neurons Supplying the Subcutaneous, Perirenal and Mesentery Fat Tissue Depots in the Pig. Acta Neurobiol Exp (Wars) (2002) 62(4):227–34. 10.55782/ane-2002-1439 12659288

[16] Hausman GJ. Anatomical and Enzyme Histochemical Differentiation of Adipose Tissue. Int J Obes (1985) 9 Suppl 1:1–6. 3934090

[17] Notohamiprodjo M Goepfert M Will S Lorbeer R Schick F Rathmann W. Renal and Renal Sinus Fat Volumes as Quantified by Magnetic Resonance Imaging in Subjects With Prediabetes, Diabetes, and Normal Glucose Tolerance. PloS One (2020) 15(2):e0216635. 10.1371/journal.pone.0216635 32074103 PMC7029849

[18] Spit KA Muskiet MHA Tonneijck L Smits MM Kramer MHH Joles JA. Renal Sinus Fat and Renal Hemodynamics: A Cross-Sectional Analysis. Magma (2020) 33(1):73–80. 10.1007/s10334-019-00773-z 31471702 PMC7021744

[19] Hamer DW Santer RM. Anatomy and Blood Supply of the Coeliac-Superior Mesenteric Ganglion Complex of the Rat. Anat Embryol (Berl) (1981) 162(3):353–62. 10.1007/BF00299978 7270906

[20] Shoja MM Tubbs RS Loukas M Shokouhi G Ghabili K Agutter PS. The Sub-Peritoneal Arterial Plexus of Sir William Turner. Ann Anat (2010) 192(4):194–8. 10.1016/j.aanat.2010.05.001 20634049

[21] Jespersen NZ Feizi A Andersen ES Heywood S Hattel HB Daugaard S. Heterogeneity in the Perirenal Region of Humans Suggests Presence of Dormant Brown Adipose Tissue That Contains Brown Fat Precursor Cells. Mol Metab (2019) 24:30–43. 10.1016/j.molmet.2019.03.005 31079959 PMC6531810

[22] Wu NN Zhang CH Lee HJ Ma Y Wang X Ma XJ. Brown Adipogenic Potential of Brown Adipocytes and Peri-Renal Adipocytes From Human Embryo. Sci Rep (2016) 6:39193. 10.1038/srep39193 27982067 PMC5159842

[23] Grigoraș A Balan RA Căruntu I-D Giușcă SE Lozneanu L Avadanei RE. Perirenal Adipose Tissue—Current Knowledge and Future Opportunities. J Clin Med (2021) 10(6):1291. 10.3390/jcm10061291 33800984 PMC8004049

[24] Huang N Mao EW Hou NN Liu YP Han F Sun XD. Novel Insight Into Perirenal Adipose Tissue: A Neglected Adipose Depot Linking Cardiovascular and Chronic Kidney Disease. World J Diabetes (2020) 11(4):115–25. 10.4239/wjd.v11.i4.115 PMC7156295 32313610

[25] Efremova A Senzacqua M Venema W Isakov E Di Vincenzo A Zingaretti MC. A Large Proportion of Mediastinal and Perirenal Visceral Fat of Siberian Adult People is Formed by UCP1 Immunoreactive Multilocular and Paucilocular Adipocytes. J Physiol Biochem (2020) 76(2):185–92. 10.1007/s13105-019-00721-4 31853729

[26] Svensson PA Lindberg K Hoffmann JM Taube M Pereira MJ Mohsen-Kanson T. Characterization of Brown Adipose Tissue in the Human Perirenal Depot. Obes (Silver Spring) (2014) 22(8):1830–7. 10.1002/oby.20765 24753268

[27] Baer PC Koch B Hickmann E Schubert R Cinatl J Jr. Hauser IA. Isolation, Characterization, Differentiation and Immunomodulatory Capacity of Mesenchymal Stromal/Stem Cells From Human Perirenal Adipose Tissue. Cells (2019) 8(11):1–17. 10.3390/cells8111346 PMC6928994 31671899

[28] Eisner BH Zargooshi J Berger AD Cooperberg MR Doyle SM Sheth S. Gender Differences in Subcutaneous and Perirenal Fat Distribution. Surg Radiol Anat (2010) 32(9):879–82. 10.1007/s00276-010-0692-7 20607260

[29] Favre G Grangeon-Chapon C Raffaelli C François-Chalmin F Iannelli A Esnault V. Perirenal Fat Thickness Measured With Computed Tomography is a Reliable Estimate of Perirenal Fat Mass. PloS One (2017) 12(4):e0175561. 10.1371/journal.pone.0175561 28423008 PMC5396915

[30] van den Beukel JC Grefhorst A Hoogduijn MJ Steenbergen J Mastroberardino PG Dor FJ. Women Have More Potential to Induce Browning of Perirenal Adipose Tissue Than Men. Obes (Silver Spring) (2015) 23(8):1671–9. 10.1002/oby.21166 26179979

[31] Chen J Chen JK Nagai K Plieth D Tan M Lee TC. EGFR Signaling Promotes Tgfbeta-Dependent Renal Fibrosis. J Am Soc Nephrol (2012) 23(2):215–24. 10.1681/ASN.2011070645 PMC3269185 22095949

[32] Geraci G Zammuto MM Mattina A Zanoli L Geraci C Granata A. Para-Perirenal Distribution of Body Fat is Associated With Reduced Glomerular Filtration Rate Regardless of Other Indices of Adiposity in Hypertensive Patients. J Clin Hypertens (Greenwich) (2018) 20(10):1438–46. 10.1111/jch.13366 PMC8030936 30218482

[33] Esler M Straznicky N Eikelis N Masuo K Lambert G Lambert E. Mechanisms of Sympathetic Activation in Obesity-Related Hypertension. Hypertension (2006) 48(5):787–96. 10.1161/01.HYP.0000242642.42177.49 17000932

[34] Messina G De Luca V Viggiano A Ascione A Iannaccone T Chieffi S. Autonomic Nervous System in the Control of Energy Balance and Body Weight: Personal Contributions. Neurol Res Int (2013) 2013:639280. 10.1155/2013/639280 23691314 PMC3649682

[35] Young HM Cane KN Anderson CR. Development of the Autonomic Nervous System: A Comparative View. Auton Neurosci (2011) 165(1):10–27. 10.1016/j.autneu.2010.03.002 20346736

[36] Grassi G Biffi A Seravalle G Trevano FQ Dell’Oro R Corrao G. Sympathetic Neural Overdrive in the Obese and Overweight State. Hypertension (2019) 74(2):349–58. 10.1161/HYPERTENSIONAHA.119.12885 31203727

[37] Hall JE da Silva AA do Carmo JM Dubinion J Hamza S Munusamy S. Obesity-Induced Hypertension: Role of Sympathetic Nervous System, Leptin, and Melanocortins. J Biol Chem (2010) 285(23):17271–6. 10.1074/jbc.R110.113175 PMC2878489 20348094

[38] Mancia G Grassi G Giannattasio C Seravalle G. Sympathetic Activation in the Pathogenesis of Hypertension and Progression of Organ Damage. Hypertension (1999) 34(4 Pt 2):724–8. 10.1161/01.HYP.34.4.724 10523349

[39] Messerli FH Williams B Ritz E. Essential Hypertension. Lancet (2007) 370(9587):591–603. 10.1016/S0140-6736(07)61299-9 17707755

[40] Grassi G Mark A Esler M. The Sympathetic Nervous System Alterations in Human Hypertension. Circ Res (2015) 116(6):976–90. 10.1161/CIRCRESAHA.116.303604 PMC4367954 25767284

[41] Zeng W Pirzgalska RM Pereira MM Kubasova N Barateiro A Seixas E. Sympathetic Neuro-Adipose Connections Mediate Leptin-Driven Lipolysis. Cell (2015) 163(1):84–94. 10.1016/j.cell.2015.08.055 26406372 PMC7617198

[42] Cao Q Jing J Cui X Shi H Xue B. Sympathetic Nerve Innervation is Required for Beigeing in White Fat. Physiol Rep (2019) 7(6):e14031. 10.14814/phy2.14031 30873754 PMC6418318

[43] Sipe LM Yang C Ephrem J Garren E Hirsh J Deppmann CD. Differential Sympathetic Outflow to Adipose Depots is Required for Visceral Fat Loss in Response to Calorie Restriction. Nutr Diabetes (2017) 7(4):e260. 10.1038/nutd.2017.13 28394360 PMC5436093

[44] Shi Z Chen WW Xiong XQ Han Y Zhou YB Zhang F. Sympathetic Activation by Chemical Stimulation of White Adipose Tissues in Rats. J Appl Physiol (1985) (2012) 112(6):1008–14. 10.1152/japplphysiol.01164.2011 22223453

[45] Bartness TJ Shrestha YB Vaughan CH Schwartz GJ Song CK. Sensory and Sympathetic Nervous System Control of White Adipose Tissue Lipolysis. Mol Cell Endocrinol (2010) 318(1-2):34–43. 10.1016/j.mce.2009.08.031 19747957 PMC2826518

[46] Niijima A. Afferent Signals From Leptin Sensors in the White Adipose Tissue of the Epididymis, and Their Reflex Effect in the Rat. J Auton Nerv Syst (1998) 73(1):19–25. 10.1016/S0165-1838(98)00109-X 9808367

[47] Xiong XQ Chen WW Han Y Zhou YB Zhang F Gao XY. Enhanced Adipose Afferent Reflex Contributes to Sympathetic Activation in Diet-Induced Obesity Hypertension. Hypertension (2012) 60(5):1280–6. 10.1161/HYPERTENSIONAHA.112.198002 23033372

[48] Ding L Kang Y Dai HB Wang FZ Zhou H Gao Q. Adipose Afferent Reflex is Enhanced by Tnfα in Paraventricular Nucleus Through NADPH Oxidase-Dependent ROS Generation in Obesity-Related Hypertensive Rats. J Transl Med (2019) 17(1):256. 10.1186/s12967-019-2006-0 31391086 PMC6686415

[49] Dalmasso C Leachman JR Osborn JL Loria AS. Sensory Signals Mediating High Blood Pressure *via* Sympathetic Activation: Role of Adipose Afferent Reflex. Am J Physiol Regul Integr Comp Physiol (2020) 318(2):R379–89. 10.1152/ajpregu.00079.2019 PMC7052594 31868518

[50] Tanida M Iwashita S Ootsuka Y Terui N Suzuki M. Leptin Injection Into White Adipose Tissue Elevates Renal Sympathetic Nerve Activity Dose-Dependently Through the Afferent Nerves Pathway in Rats. Neurosci Lett (2000) 293(2):107–10. 10.1016/S0304-3940(00)01490-7 11027845

[51] Cao Q Zhang J Yu Q Wang J Dai M Zhang Y. Carotid Baroreceptor Stimulation in Obese Rats Affects White and Brown Adipose Tissues Differently in Metabolic Protection. J Lipid Res (2019) 60(7):1212–24. 10.1194/jlr.M091256 PMC6602135 31126973

[52] Cao Q Liu L Hu Y Jiang N Wang Y Chen J. Irradiation of Carotid Baroreceptor With Low-Intensity Pulsed Ultrasound Exerts Different Metabolic Protection in Perirenal, Epididymal White Adipose Tissue and Interscapular Brown Adipose Tissue of Obese Rats. FASEB J (2020) 34(11):15431–47. 10.1096/fj.202001550R 32954572

[53] Puar T van Berkel A Gotthardt M Havekes B Hermus AR Lenders JW. Genotype-Dependent Brown Adipose Tissue Activation in Patients With Pheochromocytoma and Paraganglioma. J Clin Endocrinol Metab (2016) 101(1):224–32. 10.1210/jc.2015-3205 26574955

[54] Pereira MP Ferreira LAA da Silva FHS Christoffolete MA Metsios GS Chaves VE. A Low-Protein, High-Carbohydrate Diet Increases Browning in Perirenal Adipose Tissue But Not in Inguinal Adipose Tissue. Nutrition (2017) 42:37–45. 10.1016/j.nut.2017.05.007 28870477

[55] Warner A Kjellstedt A Carreras A Böttcher G Peng XR Seale P. Activation of β3-Adrenoceptors Increases *in Vivo* Free Fatty Acid Uptake and Utilization in Brown But Not White Fat Depots in High-Fat-Fed Rats. Am J Physiol Endocrinol Metab (2016) 311(6):E901–10. 10.1152/ajpendo.00204.2016 PMC5183882 27780820

[56] Mancuso P. The Role of Adipokines in Chronic Inflammation. Immunotargets Ther (2016) 5:47–56. 10.2147/ITT.S73223 27529061 PMC4970637

[57] Herold J Kalucka J. Angiogenesis in Adipose Tissue: The Interplay Between Adipose and Endothelial Cells. Front Physiol (2020) 11:624903. 10.3389/fphys.2020.624903 33633579 PMC7900516

[58] Janochova K Haluzik M Buzga M. Visceral Fat and Insulin Resistance - What We Know? BioMed Pap Med Fac Univ Palacky Olomouc Czech Repub (2019) 163(1):19–27. 10.5507/bp.2018.062 30398218

[59] Chau YY Bandiera R Serrels A Martínez-Estrada OM Qing W Lee M. Visceral and Subcutaneous Fat Have Different Origins and Evidence Supports a Mesothelial Source. Nat Cell Biol (2014) 16(4):367–75. 10.1038/ncb2922 PMC4060514 24609269

[60] Lau WB Ohashi K Wang Y Ogawa H Murohara T Ma XL. Role of Adipokines in Cardiovascular Disease. Circ J (2017) 81(7):920–8. 10.1253/circj.CJ-17-0458 28603178

[61] AlZaim I Hammoud SH Al-Koussa H Ghazi A Eid AH El-Yazbi AF. Adipose Tissue Immunomodulation: A Novel Therapeutic Approach in Cardiovascular and Metabolic Diseases. Front Cardiovasc Med (2020) 7:602088. 10.3389/fcvm.2020.602088 33282920 PMC7705180

[62] Triantafyllou GA Paschou SA Mantzoros CS. Leptin and Hormones: Energy Homeostasis. Endocrinol Metab Clin North Am (2016) 45(3):633–45. 10.1016/j.ecl.2016.04.012 27519135

[63] Yadav A Kataria MA Saini V Yadav A. Role of Leptin and Adiponectin in Insulin Resistance. Clin Chim Acta (2013) 417:80–4. 10.1016/j.cca.2012.12.007 23266767

[64] Fukui A Ikebe-Ebata Y Kondo H Saito S Aoki K Fukunaga N. Hyperleptinemia Exacerbates High-Fat Diet-Mediated Atrial Fibrosis and Fibrillation. J Cardiovasc Electrophysiol (2017) 28(6):702–10. 10.1111/jce.13200 28257569

[65] Pretz D Le Foll C Rizwan MZ Lutz TA Tups A. Hyperleptinemia as a Contributing Factor for the Impairment of Glucose Intolerance in Obesity. FASEB J (2021) 35(2):e21216. 10.1096/fj.202001147R 33230896

[66] Galletti F D’Elia L De Palma D Russo O Barba G Siani A. Hyperleptinemia is Associated With Hypertension, Systemic Inflammation and Insulin Resistance in Overweight But Not in Normal Weight Men. Nutr Metab Cardiovasc Dis (2012) 22(3):300–6. 10.1016/j.numecd.2011.05.007 21920718

[67] Li H Li M Liu P Wang Y Zhang H Li H. Telmisartan Ameliorates Nephropathy in Metabolic Syndrome by Reducing Leptin Release From Perirenal Adipose Tissue. Hypertension (2016) 68(2):478–90. 10.1161/HYPERTENSIONAHA.116.07008 27296996

[68] Turer AT Scherer PE. Adiponectin: Mechanistic Insights and Clinical Implications. Diabetologia (2012) 55(9):2319–26. 10.1007/s00125-012-2598-x 22688349

[69] Díez JJ Iglesias P. The Role of the Novel Adipocyte-Derived Protein Adiponectin in Human Disease: An Update. Mini Rev Med Chem (2010) 10(9):856–69. 10.2174/138955710791608325 20482500

[70] Ukkola O Santaniemi M. Adiponectin: A Link Between Excess Adiposity and Associated Comorbidities? J Mol Med (Berl) (2002) 80(11):696–702. 10.1007/s00109-002-0378-7 12436346

[71] La Russa D Marrone A Mandalà M Macirella R Pellegrino D. Antioxidant/Anti-Inflammatory Effects of Caloric Restriction in an Aged and Obese Rat Model: The Role of Adiponectin. Biomedicines (2020) 8(12):1–10. 10.3390/biomedicines8120532 PMC7761007 33255520

[72] Zhao Y Gao P Sun F Li Q Chen J Yu H. Sodium Intake Regulates Glucose Homeostasis Through the Pparδ/Adiponectin-Mediated SGLT2 Pathway. Cell Metab (2016) 23(4):699–711. 10.1016/j.cmet.2016.02.019 27053360

[73] Santos SH Braga JF Mario EG Pôrto LC Rodrigues-Machado Mda G Murari A. Improved Lipid and Glucose Metabolism in Transgenic Rats With Increased Circulating Angiotensin-(1-7). Arterioscler Thromb Vasc Biol (2010) 30(5):953–61. 10.1161/ATVBAHA.109.200493 20203301

[74] Dünner N Quezada C Berndt FA Cánovas J Rojas CV. Angiotensin II Signaling in Human Preadipose Cells: Participation of ERK1,2-Dependent Modulation of Akt. PloS One (2013) 8(10):e75440. 10.1371/journal.pone.0075440 24098385 PMC3788799

[75] Yvan-Charvet L Quignard-Boulangé A. Role of Adipose Tissue Renin-Angiotensin System in Metabolic and Inflammatory Diseases Associated With Obesity. Kidney Int (2011) 79(2):162–8. 10.1038/ki.2010.391 20944545

[76] Menikdiwela KR Ramalingam L Rasha F Wang S Dufour JM Kalupahana NS. Autophagy in Metabolic Syndrome: Breaking the Wheel by Targeting the Renin-Angiotensin System. Cell Death Dis (2020) 11(2):87. 10.1038/s41419-020-2275-9 32015340 PMC6997396

[77] Santos RA Ferreira AJ Verano-Braga T Bader M. Angiotensin-Converting Enzyme 2, Angiotensin-(1-7) and Mas: New Players of the Renin-Angiotensin System. J Endocrinol (2013) 216(2):R1–17. 10.1530/JOE-12-0341 23092879

[78] Engeli S Schling P Gorzelniak K Boschmann M Janke J Ailhaud G. The Adipose-Tissue Renin-Angiotensin-Aldosterone System: Role in the Metabolic Syndrome? Int J Biochem Cell Biol (2003) 35(6):807–25. 10.1016/S1357-2725(02)00311-4 12676168

[79] Hainault I Nebout G Turban S Ardouin B Ferré P Quignard-Boulangé A. Adipose Tissue-Specific Increase in Angiotensinogen Expression and Secretion in the Obese (Fa/Fa) Zucker Rat. Am J Physiol Endocrinol Metab (2002) 282(1):E59–66. 10.1152/ajpendo.2002.282.1.E59 11739084

[80] Barton M Carmona R Morawietz H d’Uscio LV Goettsch W Hillen H. Obesity is Associated With Tissue-Specific Activation of Renal Angiotensin-Converting Enzyme *in Vivo*: Evidence for a Regulatory Role of Endothelin. Hypertension (2000) 35(1 Pt 2):329–36. 10.1161/01.HYP.35.1.329 10642320

[81] Schling P Schäfer T. Human Adipose Tissue Cells Keep Tight Control on the Angiotensin II Levels in Their Vicinity. J Biol Chem (2002) 277(50):48066–75. 10.1074/jbc.M204058200 12196514

[82] Crandall DL Busler DE McHendry-Rinde B Groeling TM Kral JG. Autocrine Regulation of Human Preadipocyte Migration by Plasminogen Activator Inhibitor-1. J Clin Endocrinol Metab (2000) 85(7):2609–14. 10.1210/jcem.85.7.6678 10902815

[83] Katovich MJ Pachori A. Effects of Inhibition of the Renin-Angiotensin System on the Cardiovascular Actions of Insulin. Diabetes Obes Metab (2000) 2(1):3–14. 10.1046/j.1463-1326.2000.00044.x 11220351

[84] Bloem LJ Manatunga AK Tewksbury DA Pratt JH. The Serum Angiotensinogen Concentration and Variants of the Angiotensinogen Gene in White and Black Children. J Clin Invest (1995) 95(3):948–53. 10.1172/JCI117803 PMC441426 7883995

[85] Cooper R Forrester T Ogunbiyi O Muffinda J. Angiotensinogen Levels and Obesity in Four Black Populations. ICSHIB Investigators. J Hypertens (1998) 16(5):571–5. 10.1097/00004872-199816050-00003 9797167

[86] Goodfriend TL Kelley DE Goodpaster BH Winters SJ. Visceral Obesity and Insulin Resistance are Associated With Plasma Aldosterone Levels in Women. Obes Res (1999) 7(4):355–62. 10.1002/j.1550-8528.1999.tb00418.x 10440591

[87] Umemura S Nyui N Tamura K Hibi K Yamaguchi S Nakamaru M. Plasma Angiotensinogen Concentrations in Obese Patients. Am J Hypertens (1997) 10(6):629–33. 10.1016/S0895-7061(97)00053-8 9194508

[88] van Harmelen V Elizalde M Ariapart P Bergstedt-Lindqvist S Reynisdottir S Hoffstedt J. The Association of Human Adipose Angiotensinogen Gene Expression With Abdominal Fat Distribution in Obesity. Int J Obes Relat Metab Disord (2000) 24(6):673–8. 10.1038/sj.ijo.0801217 10878672

[89] Van Harmelen V Ariapart P Hoffstedt J Lundkvist I Bringman S Arner P. Increased Adipose Angiotensinogen Gene Expression in Human Obesity. Obes Res (2000) 8(4):337–41. 10.1038/oby.2000.40 10933310

[90] Gorzelniak K Engeli S Janke J Luft FC Sharma AM. Hormonal Regulation of the Human Adipose-Tissue Renin-Angiotensin System: Relationship to Obesity and Hypertension. J Hypertens (2002) 20(5):965–73. 10.1097/00004872-200205000-00032 12011658

[91] Faloia E Gatti C Camilloni MA Mariniello B Sardu C Garrapa GG. Comparison of Circulating and Local Adipose Tissue Renin-Angiotensin System in Normotensive and Hypertensive Obese Subjects. J Endocrinol Invest (2002) 25(4):309–14. 10.1007/BF03344010 12030600

[92] Hung WW Hsieh TJ Lin T Chou PC Hsiao PJ Lin KD. Blockade of the Renin-Angiotensin System Ameliorates Apelin Production in 3T3-L1 Adipocytes. Cardiovasc Drugs Ther (2011) 25(1):3–12. 10.1007/s10557-010-6274-4 21161354

[93] Skurk T van Harmelen V Blum WF Hauner H. Angiotensin II Promotes Leptin Production in Cultured Human Fat Cells by an ERK1/2-Dependent Pathway. Obes Res (2005) 13(6):969–73. 10.1038/oby.2005.113 15976138

[94] Frigolet ME Torres N Tovar AR. The Renin-Angiotensin System in Adipose Tissue and its Metabolic Consequences During Obesity. J Nutr Biochem (2013) 24(12):2003–15. 10.1016/j.jnutbio.2013.07.002 24120291

[95] Nisoli E Briscini L Giordano A Tonello C Wiesbrock SM Uysal KT. Tumor Necrosis Factor Alpha Mediates Apoptosis of Brown Adipocytes and Defective Brown Adipocyte Function in Obesity. Proc Natl Acad Sci USA (2000) 97(14):8033–8. 10.1073/pnas.97.14.8033 PMC16665 10884431

[96] Simons PJ van den Pangaart PS Aerts JM Boon L. Pro-Inflammatory Delipidizing Cytokines Reduce Adiponectin Secretion From Human Adipocytes Without Affecting Adiponectin Oligomerization. J Endocrinol (2007) 192(2):289–99. 10.1677/JOE-06-0047 17283229

[97] Oh YB Kim JH Park BM Park BH Kim SH. Captopril Intake Decreases Body Weight Gain *via* Angiotensin-(1-7). Peptides (2012) 37(1):79–85. 10.1016/j.peptides.2012.06.005 22743141

[98] Than A Leow MK Chen P. Control of Adipogenesis by the Autocrine Interplays Between Angiotensin 1-7/Mas Receptor and Angiotensin II/AT1 Receptor Signaling Pathways. J Biol Chem (2013) 288(22):15520–31. 10.1074/jbc.M113.459792 PMC3668713 23592774

[99] de Macedo SM Guimarares TA Guimarares TA Andrade JMO Andrade JM Guimaraes ALS. Angiotensin Converting Enzyme 2 Activator (DIZE) Modulates Metabolic Profiles in Mice, Decreasing Lipogenesis. Protein Pept Lett (2015) 22(4):332–40. 10.2174/0929866522666150209125401 25666042

[100] Santos SH Andrade JM Fernandes LR Fernandes LR Sinisterra RDM Sinisterra RD. Oral Angiotensin-(1-7) Prevented Obesity and Hepatic Inflammation by Inhibition of Resistin/TLR4/MAPK/NF-κb in Rats Fed With High-Fat Diet. Peptides (2013) 46:47–52. 10.1016/j.peptides.2013.05.010 23714175

[101] Cabandugama PK Gardner MJ Sowers JR. The Renin Angiotensin Aldosterone System in Obesity and Hypertension: Roles in the Cardiorenal Metabolic Syndrome. Med Clin North Am (2017) 101(1):129–37. 10.1016/j.mcna.2016.08.009 PMC5125542 27884224

[102] Schütten MT Houben AJ de Leeuw PW Stehouwer CD. The Link Between Adipose Tissue Renin-Angiotensin-Aldosterone System Signaling and Obesity-Associated Hypertension. Physiology (2017) 32(3):197–209. 10.1152/physiol.00037.2016 28404736

[103] Sarzani R Marcucci P Salvi F Salvi F Bordicchia M Bordicchia M. Angiotensin II Stimulates and Atrial Natriuretic Peptide Inhibits Human Visceral Adipocyte Growth. Int J Obes (Lond) (2008) 32(2):259–67. 10.1038/sj.ijo.0803724 17878892

[104] Mori J Patel VB Ramprasath T Alrob OA DesAulniers J Scholey JW. Angiotensin 1–7 Mediates Renoprotection Against Diabetic Nephropathy by Reducing Oxidative Stress, Inflammation, and Lipotoxicity. Am J Physiology-Renal Physiol (2014) 306(8):F812–21. 10.1152/ajprenal.00655.2013 24553436

[105] Pillon NJ Loos RJ Marshall SM Zierath JR. Metabolic Consequences of Obesity and Type 2 Diabetes: Balancing Genes and Environment for Personalized Care. Cell (2021) 184(6):1530–44. 10.1016/j.cell.2021.02.012 PMC9191863 33675692

[106] Chait A den Hartigh LJ. Adipose Tissue Distribution, Inflammation and its Metabolic Consequences, Including Diabetes and Cardiovascular Disease. Front Cardiovasc Med (2020) 7:1–41. 10.3389/fcvm.2020.00022 32158768 PMC7052117

[107] Santoro A McGraw TE Kahn BB. Insulin Action in Adipocytes, Adipose Remodeling, and Systemic Effects. Cell Metab (2021) 33(4):748–57. 10.1016/j.cmet.2021.03.019 PMC8078167 33826917

[108] Lee YS J-w K Osborne O Sasik R Schenk S Chen A. Increased Adipocyte O2 Consumption Triggers HIF-1α, Causing Inflammation and Insulin Resistance in Obesity. Cell (2014) 157(6):1339–52. 10.1016/j.cell.2014.05.012 PMC4114226 24906151

[109] Pedersen DJ Guilherme A Danai LV Heyda L Matevossian A Cohen J. A Major Role of Insulin in Promoting Obesity-Associated Adipose Tissue Inflammation. Mol Metab (2015) 4(7):507–18. 10.1016/j.molmet.2015.04.003 PMC4481426 26137438

[110] Kane H Lynch L. Innate Immune Control of Adipose Tissue Homeostasis. Trends Immunol (2019) 40(9):857–72. 10.1016/j.it.2019.07.006 31399336

[111] Choe SS Huh JY Hwang IJ Kim JI Kim JB. Adipose Tissue Remodeling: Its Role in Energy Metabolism and Metabolic Disorders. Front Endocrinol (Lausanne) (2016) 7:30. 10.3389/fendo.2016.00030 27148161 PMC4829583

[112] Engin A. The Pathogenesis of Obesity-Associated Adipose Tissue Inflammation. Obes lipotoxicity (2017) 960:221–45. 10.1007/978-3-319-48382-5_9 28585201

[113] Legrand-Poels S Esser N L’homme L Scheen A Paquot N Piette J. Free Fatty Acids as Modulators of the NLRP3 Inflammasome in Obesity/Type 2 Diabetes. Biochem Pharmacol (2014) 92(1):131–41. 10.1016/j.bcp.2014.08.013 25175736

[114] Morigny P Houssier M Mouisel E Langin D. Adipocyte Lipolysis and Insulin Resistance. Biochimie (2016) 125:259–66. 10.1016/j.biochi.2015.10.024 26542285

[115] Kuroda M Sakaue H. Adipocyte Death and Chronic Inflammation in Obesity. J Med Invest (2017) 64(3.4):193–6. 10.2152/jmi.64.193 28954980

[116] Crewe C An YA Scherer PE. The Ominous Triad of Adipose Tissue Dysfunction: Inflammation, Fibrosis, and Impaired Angiogenesis. J Clin Invest (2017) 127(1):74–82. 10.1172/JCI88883 28045400 PMC5199684

[117] Hussain MF Roesler A Kazak L. Regulation of Adipocyte Thermogenesis: Mechanisms Controlling Obesity. FEBS J (2020) 287(16):3370–85. 10.1111/febs.15331 32301220

[118] Cannon B Nedergaard J. What Ignites UCP1? Cell Metab (2017) 26(5):697–8. 10.1016/j.cmet.2017.10.012 29117542

[119] Roesler A Kazak L. UCP1-Independent Thermogenesis. Biochem J (2020) 477(3):709–25. 10.1042/BCJ20190463 32059055

[120] Kazak L Cohen P. Creatine Metabolism: Energy Homeostasis, Immunity and Cancer Biology. Nat Rev Endocrinol (2020) 16(8):421–36. 10.1038/s41574-020-0365-5 32493980

[121] Rahbani JF Roesler A Hussain MF Samborska B Dykstra CB Tsai L. Creatine Kinase B Controls Futile Creatine Cycling in Thermogenic Fat. Nature (2021) 590(7846):480–5. 10.1038/s41586-021-03221-y PMC8647628 33597756

[122] Bertholet AM Kazak L Chouchani ET Bogaczyńska MG Paranjpe I Wainwright GL. Mitochondrial Patch Clamp of Beige Adipocytes Reveals UCP1-Positive and UCP1-Negative Cells Both Exhibiting Futile Creatine Cycling. Cell Metab (2017) 25(4):811–22.e4. 10.1016/j.cmet.2017.03.002 28380374 PMC5448977

[123] Kazak L Chouchani ET Jedrychowski MP Erickson BK Shinoda K Cohen P. A Creatine-Driven Substrate Cycle Enhances Energy Expenditure and Thermogenesis in Beige Fat. Cell (2015) 163(3):643–55. 10.1016/j.cell.2015.09.035 PMC4656041 26496606

[124] Kazak L Chouchani ET Lu GZ Jedrychowski MP Bare CJ Mina AI. Genetic Depletion of Adipocyte Creatine Metabolism Inhibits Diet-Induced Thermogenesis and Drives Obesity. Cell Metab (2017) 26(4):660–71. e3. 10.1016/j.cmet.2017.08.009 28844881 PMC5629120

[125] Kazak L Rahbani JF Samborska B Lu GZ Jedrychowski MP Lajoie M. Ablation of Adipocyte Creatine Transport Impairs Thermogenesis and Causes Diet-Induced Obesity. Nat Metab (2019) 1(3):360–70. 10.1038/s42255-019-0035-x PMC6544051 31161155

[126] Shabalina IG Petrovic N de Jong JM Kalinovich AV Cannon B Nedergaard J. UCP1 in Brite/Beige Adipose Tissue Mitochondria is Functionally Thermogenic. Cell Rep (2013) 5(5):1196–203. 10.1016/j.celrep.2013.10.044 24290753

[127] Vijgen GHEJ Sparks LM Bouvy ND Schaart G Hoeks J van Marken Lichtenbelt WD. Increased Oxygen Consumption in Human Adipose Tissue From the “Brown Adipose Tissue” Region. J Clin Endocrinol Metab (2013) 98(7):E1230–E4. 10.1210/jc.2013-1348 23783102

[128] Schneider K Valdez J Nguyen J Vawter M Galke B Kurtz TW. Increased Energy Expenditure, Ucp1 Expression, and Resistance to Diet-Induced Obesity in Mice Lacking Nuclear Factor-Erythroid-2-Related Transcription Factor-2 (Nrf2). J Biol Chem (2016) 291(14):7754–66. 10.1074/jbc.M115.673756 PMC4817199 26841864

[129] Manno C Campobasso N Nardecchia A Triggiani V Zupo R Gesualdo L. Relationship of Para- and Perirenal Fat and Epicardial Fat With Metabolic Parameters in Overweight and Obese Subjects. Eat Weight Disord (2019) 24(1):67–72. 10.1007/s40519-018-0532-z 29956099

[130] Roever L Resende ES Veloso FC Diniz AL Penha-Silva N Casella-Filho A. Perirenal Fat and Association With Metabolic Risk Factors: The Uberlândia Heart Study. Med (Baltimore) (2015) 94(38):e1105. 10.1097/MD.0000000000001105 26402796

[131] Li X Liu J Wang G Yu J Sheng Y Wang C. Determination of UCP1 Expression in Subcutaneous and Perirenal Adipose Tissues of Patients With Hypertension. Endocrine (2015) 50(2):413–23. 10.1007/s12020-015-0572-3 25784389

[132] Fromme T Klingenspor M. Uncoupling Protein 1 Expression and High-Fat Diets. Am J Physiol Regulatory Integr Comp Physiol (2011) 300(1):R1–8. 10.1152/ajpregu.00411.2010 21048077

[133] Fedorenko A Lishko PV Kirichok Y. Mechanism of Fatty-Acid-Dependent UCP1 Uncoupling in Brown Fat Mitochondria. Cell (2012) 151(2):400–13. 10.1016/j.cell.2012.09.010 PMC3782081 23063128

[134] Luijten IHN Feldmann HM Gv E Cannon B Nedergaard J. In the Absence of UCP1-Mediated Diet-Induced Thermogenesis, Obesity is Augmented Even in the Obesity-Resistant 129S Mouse Strain. Am J Physiol-Endocrinol Metab (2019) 316(5):E729–E40. 10.1152/ajpendo.00020.2019 PMC6580168 30807213

[135] Chouchani ET Kazak L Jedrychowski MP Lu GZ Erickson BK Szpyt J. Mitochondrial ROS Regulate Thermogenic Energy Expenditure and Sulfenylation of UCP1. Nature (2016) 532(7597):112–6. 10.1038/nature17399 PMC5549630 27027295

[136] Li X Wang G Liu J Ding G. Increased UCP1 Expression in the Perirenal Adipose Tissue of Patients With Renal Cell Carcinoma. Oncol Rep (2019) 42(5):1972–80. 10.3892/or.2019.7306 PMC6775817 31545449

[137] Rafeh R Viveiros A Oudit GY El-Yazbi AF. Targeting Perivascular and Epicardial Adipose Tissue Inflammation: Therapeutic Opportunities for Cardiovascular Disease. Clin Sci (2020) 134(7):827–51. 10.1042/CS20190227 32271386

[138] Wu C Zhang H Zhang J Xie C Fan C Zhang H. Inflammation and Fibrosis in Perirenal Adipose Tissue of Patients With Aldosterone-Producing Adenoma. Endocrinology (2018) 159(1):227–37. 10.1210/en.2017-00651 29059354

[139] Wu C Zhang H Zhang J Zhang H Zeng Y Fang S. Increased Oxidative Stress, Inflammation and Fibrosis in Perirenal Adipose Tissue of Patients With Cortisol-Producing Adenoma. Adipocyte (2019) 8(1):347–56. 10.1080/21623945.2019.1690834 PMC6948963 31718404

[140] Ahmad F Soelaiman IN Ramli ES Hooi TM Suhaimi FH. Histomorphometric Changes in the Perirenal Adipocytes of Adrenalectomized Rats Treated With Dexamethasone. Clinics (Sao Paulo) (2011) 66(5):849–53. 10.1590/S1807-59322011000500023 PMC3109386 21789391

[141] Roerink S Wagenmakers M Langenhuijsen JF Ballak DB Rooijackers HMM d’Ancona FC. Increased Adipocyte Size, Macrophage Infiltration, and Adverse Local Adipokine Profile in Perirenal Fat in Cushing’s Syndrome. Obes (Silver Spring) (2017) 25(8):1369–74. 10.1002/oby.21887 28594137

[142] Nagano G Ohno H Oki K Kobuke K Shiwa T Yoneda M. Activation of Classical Brown Adipocytes in the Adult Human Perirenal Depot is Highly Correlated With PRDM16-EHMT1 Complex Expression. PloS One (2015) 10(3):e0122584. 10.1371/journal.pone.0122584 25812118 PMC4374757

[143] Weisinger JR Kempson RL Eldridge FL Swenson RS. The Nephrotic Syndrome: A Complication of Massive Obesity. Ann Intern Med (1974) 81(4):440–7. 10.7326/0003-4819-81-4-440 4416380

[144] Ejerblad E Fored CM Lindblad P Fryzek J McLaughlin JK Nyrén O. Obesity and Risk for Chronic Renal Failure. J Am Soc Nephrol (2006) 17(6):1695–702. 10.1681/ASN.2005060638 16641153

[145] Sun X Han F Miao W Hou N Cao Z Zhang G. Sonographic Evaluation of Para- and Perirenal Fat Thickness is an Independent Predictor of Early Kidney Damage in Obese Patients. Int Urol Nephrol (2013) 45(6):1589–95. 10.1007/s11255-013-0404-4 23463155

[146] Othman M Kawar B El Nahas AM. Influence of Obesity on Progression of non-Diabetic Chronic Kidney Disease: A Retrospective Cohort Study. Nephron Clin Pract (2009) 113(1):c16–23. 10.1159/000228071 19590231

[147] Hsu CY McCulloch CE Iribarren C Darbinian J Go AS. Body Mass Index and Risk for End-Stage Renal Disease. Ann Intern Med (2006) 144(1):21–8. 10.7326/0003-4819-144-1-200601030-00006 16389251

[148] D’Marco L Salazar J Cortez M Salazar M Wettel M Lima-Martínez M. Perirenal Fat Thickness is Associated With Metabolic Risk Factors in Patients With Chronic Kidney Disease. Kidney Res Clin Pract (2019) 38(3):365–72. 10.23876/j.krcp.18.0155 PMC6727893 31357262

[149] Hall JE do Carmo JM da Silva AA Wang Z Hall ME. Obesity, Kidney Dysfunction and Hypertension: Mechanistic Links. Nat Rev Nephrol (2019) 15(6):367–85. 10.1038/s41581-019-0145-4 PMC7278043 31015582

[150] Hall JE Mouton A da Silva AA Omoto ACM Wang Z Li X. Obesity, Kidney Dysfunction and Inflammation: Interactions in Hypertension. Cardiovasc Res (2020) 117(8):1859–76. 10.1093/cvr/cvaa336 PMC8262632 33258945

[151] Li Z Woollard JR Wang S Korsmo MJ Ebrahimi B Grande JP. Increased Glomerular Filtration Rate in Early Metabolic Syndrome is Associated With Renal Adiposity and Microvascular Proliferation. Am J Physiol Renal Physiol (2011) 301(5):F1078–87. 10.1152/ajprenal.00333.2011 PMC3213905 21775485

[152] Pinto-Sietsma SJ Navis G Janssen WM de Zeeuw D Gans RO de Jong PE. A Central Body Fat Distribution is Related to Renal Function Impairment, Even in Lean Subjects. Am J Kidney Dis (2003) 41(4):733–41. 10.1016/S0272-6386(03)00020-9 12666059

[153] Bonnet F Marre M Halimi JM Stengel B Lange C Laville M. [Larger Waist Circumference is a Predictive Factor for the Occurrence of Microalbuminuria in a non-Diabetic Population]. Arch Mal Coeur Vaiss (2006) 99(7-8):660–2. 17061439

[154] Kawasaki S Aoki K Hasegawa O Numata K Tanaka K Shibata N. Sonographic Evaluation of Visceral Fat by Measuring Para- and Perirenal Fat. J Clin Ultrasound (2008) 36(3):129–33. 10.1002/jcu.20426 18027837

[155] Foster MC Hwang SJ Porter SA Massaro JM Hoffmann U Fox CS. Fatty Kidney, Hypertension, and Chronic Kidney Disease: The Framingham Heart Study. Hypertension (2011) 58(5):784–90. 10.1161/HYPERTENSIONAHA.111.175315 PMC3204377 21931075

[156] Harper W Clement M Goldenberg R Hanna A Main A Retnakaran R. Pharmacologic Management of Type 2 Diabetes. Can J Diabetes (2013) 37 Suppl 1:S61–8. 10.1016/j.jcjd.2013.01.021 24070965

[157] Klausen KP Parving HH Scharling H Jensen JS. Microalbuminuria and Obesity: Impact on Cardiovascular Disease and Mortality. Clin Endocrinol (Oxf) (2009) 71(1):40–5. 10.1111/j.1365-2265.2008.03427.x 18803675

[158] Ma S Zhu XY Eirin A Woollard JR Jordan KL Tang H. Perirenal Fat Promotes Renal Arterial Endothelial Dysfunction in Obese Swine Through Tumor Necrosis Factor-α. J Urol (2016) 195(4 Pt 1):1152–9. 10.1016/j.juro.2015.08.105 PMC4809776 26417644

[159] Ndisang JF Jadhav A Mishra M. The Heme Oxygenase System Suppresses Perirenal Visceral Adiposity, Abates Renal Inflammation and Ameliorates Diabetic Nephropathy in Zucker Diabetic Fatty Rats. PloS One (2014) 9(1):e87936. 10.1371/journal.pone.0087936 24498225 PMC3907578

[160] Boissier R François P Gondran Tellier B Meunier M Lyonnet L Simoncini S. Perirenal Adipose Tissue Displays an Age-Dependent Inflammatory Signature Associated With Early Graft Dysfunction of Marginal Kidney Transplants. Front Immunol (2020) 11:445. 10.3389/fimmu.2020.00445 32256495 PMC7089962

[161] Králová Lesná I Poledne R Fronek J Králová A Sekerková A Thieme F. Macrophage Subsets in the Adipose Tissue Could be Modified by Sex and the Reproductive Age of Women. Atherosclerosis (2015) 241(1):255–8. 10.1016/j.atherosclerosis.2015.03.018 25795161

[162] Thieme F Janousek L Fronek J Kralova A Cejkova S Kralova Lesna I. The Effect of Ectopic Fat on Graft Function After Living Kidney Transplantation. Physiol Res (2015) 64(Suppl 3):S411–7. 10.33549/physiolres.933181 26680675

[163] Hou N Han F Wang M Huang N Zhao J Liu X. Perirenal Fat Associated With Microalbuminuria in Obese Rats. Int Urol Nephrol (2014) 46(4):839–45. 10.1007/s11255-014-0656-7 24526332

[164] Fang Y Xu Y Yang Y Liu C Zhao D Ke J. The Relationship Between Perirenal Fat Thickness and Reduced Glomerular Filtration Rate in Patients With Type 2 Diabetes. J Diabetes Res (2020) 2020:6076145. 10.1155/2020/6076145 32685560 PMC7341433

[165] Liu Y Wang L Luo M Chen N Deng X He J. Inhibition of PAI-1 Attenuates Perirenal Fat Inflammation and the Associated Nephropathy in High-Fat Diet-Induced Obese Mice. Am J Physiol Endocrinol Metab (2019) 316(2):E260–e7. 10.1152/ajpendo.00387.2018 30532990

[166] Mori J Patel VB Ramprasath T Alrob OA DesAulniers J Scholey JW. Angiotensin 1-7 Mediates Renoprotection Against Diabetic Nephropathy by Reducing Oxidative Stress, Inflammation, and Lipotoxicity. Am J Physiol Renal Physiol (2014) 306(8):F812–21. 10.1152/ajprenal.00655.2013 24553436

[167] Hall JE Crook ED Jones DW Wofford MR Dubbert PM. Mechanisms of Obesity-Associated Cardiovascular and Renal Disease. Am J Med Sci (2002) 324(3):127–37. 10.1097/00000441-200209000-00003 12240710

[168] Hall ME do Carmo JM da Silva AA Juncos LA Wang Z Hall JE. Obesity, Hypertension, and Chronic Kidney Disease. Int J Nephrol Renovasc Dis (2014) 7:75–88. 10.2147/IJNRD.S39739 24600241 PMC3933708

[169] Rea DJ Heimbach JK Grande JP Textor SC Taler SJ Prieto M. Glomerular Volume and Renal Histology in Obese and non-Obese Living Kidney Donors. Kidney Int (2006) 70(9):1636–41. 10.1038/sj.ki.5001799 16955108

[170] Montani JP Carroll JF Dwyer TM Antic V Yang Z Dulloo AG. Ectopic Fat Storage in Heart, Blood Vessels and Kidneys in the Pathogenesis of Cardiovascular Diseases. Int J Obes Relat Metab Disord (2004) 28 Suppl 4:S58–65. 10.1038/sj.ijo.0802858 15592488

[171] Adeosun SO Gordon DM Weeks MF Moore KH Hall JE Hinds TD Jr.. Loss of Biliverdin Reductase-a Promotes Lipid Accumulation and Lipotoxicity in Mouse Proximal Tubule Cells. Am J Physiol Renal Physiol (2018) 315(2):F323–f31. 10.1152/ajprenal.00495.2017 PMC6139518 29631357

[172] Sun X Yu Y Han L. High FFA Levels Related to Microalbuminuria and Uncoupling of VEGF-NO Axis in Obese Rats. Int Urol Nephrol (2013) 45(4):1197–207. 10.1007/s11255-013-0428-9 23563804

[173] Katsiki N Athyros VG Mikhailidis DP. Abnormal Peri-Organ or Intra-Organ Fat (Apifat) Deposition: An Underestimated Predictor of Vascular Risk? Curr Vasc Pharmacol (2016) 14(5):432–41. 10.2174/1570161114666160722112738 27456108

[174] Guo G Morrissey J McCracken R Tolley T Liapis H Klahr S. Contributions of Angiotensin II and Tumor Necrosis Factor-Alpha to the Development of Renal Fibrosis. Am J Physiol Renal Physiol (2001) 280(5):F777–85. 10.1152/ajprenal.2001.280.5.F777 11292619

[175] Tarzi RM Cook HT Jackson I Pusey CD Lord GM. Leptin-Deficient Mice are Protected From Accelerated Nephrotoxic Nephritis. Am J Pathol (2004) 164(2):385–90. 10.1016/S0002-9440(10)63128-8 PMC1602275 14742244

[176] Zhao HL Sui Y Guan J He L Zhu X Fan RR. Fat Redistribution and Adipocyte Transformation in Uninephrectomized Rats. Kidney Int (2008) 74(4):467–77. 10.1038/ki.2008.195 18496513

[177] Lama DJ Safiullah S Yang A Okhunov Z Landman J Clayman RV. Three-Dimensional Evaluation of Perirenal Fat Volume in Patients With Nephrolithiasis. Urolithiasis (2018) 46(6):535–41. 10.1007/s00240-018-1047-9 PMC6196721 29500620

[178] García-Carrasco A Izquierdo-Lahuerta A Medina-Gómez G. The Kidney-Heart Connection in Obesity. Nephron (2021) 13:1–5. 10.1159/000515419 33849028

[179] Perk J De Backer G Gohlke H Graham I Reiner Z Verschuren M. European Guidelines on Cardiovascular Disease Prevention in Clinical Practice (Version 2012). The Fifth Joint Task Force of the European Society of Cardiology and Other Societies on Cardiovascular Disease Prevention in Clinical Practice (Constituted by Representatives of Nine Societies and by Invited Experts). Eur Heart J (2012) 33(13):1635–701. 10.1093/eurheartj/ehs092 22555213

[180] Thompson S James M Wiebe N Hemmelgarn B Manns B Klarenbach S. Cause of Death in Patients With Reduced Kidney Function. J Am Soc Nephrol (2015) 26(10):2504–11. 10.1681/ASN.2014070714 PMC4587695 25733525

[181] Webster AC Nagler EV Morton RL Masson P. Chronic Kidney Disease. Lancet (2017) 389(10075):1238–52. 10.1016/S0140-6736(16)32064-5 27887750

[182] Vanholder R Argilés A Baurmeister U Brunet P Clark W Cohen G. Uremic Toxicity: Present State of the Art. Int J Artif Organs (2001) 24(10):695–725. 10.1177/039139880102401004 11817319

[183] Manjunath G Tighiouart H Ibrahim H MacLeod B Salem DN Griffith JL. Level of Kidney Function as a Risk Factor for Atherosclerotic Cardiovascular Outcomes in the Community. J Am Coll Cardiol (2003) 41(1):47–55. 10.1016/S0735-1097(02)02663-3 12570944

[184] Briasoulis A Bakris GL. Chronic Kidney Disease as a Coronary Artery Disease Risk Equivalent. Curr Cardiol Rep (2013) 15(3):340. 10.1007/s11886-012-0340-4 23338722

[185] Menon V Gul A Sarnak MJ. Cardiovascular Risk Factors in Chronic Kidney Disease. Kidney Int (2005) 68(4):1413–8. 10.1111/j.1523-1755.2005.00551.x 16164615

[186] Booth JN 3rd Li J Zhang L Chen L Muntner P Egan B. Trends in Prehypertension and Hypertension Risk Factors in US Adults: 1999-2012. Hypertension (2017) 70(2):275–84. 10.1161/HYPERTENSIONAHA.116.09004 PMC5594566 28607131

[187] Ades PA Savage PD. Obesity in Coronary Heart Disease: An Unaddressed Behavioral Risk Factor. Prev Med (2017) 104:117–9. 10.1016/j.ypmed.2017.04.013 PMC5640469 28414064

[188] Saklayen MG. The Global Epidemic of the Metabolic Syndrome. Curr Hypertens Rep (2018) 20(2):12. 10.1007/s11906-018-0812-z 29480368 PMC5866840

[189] Piché ME Tchernof A Després JP. Obesity Phenotypes, Diabetes, and Cardiovascular Diseases. Circ Res (2020) 126(11):1477–500. 10.1161/CIRCRESAHA.120.316101 32437302

[190] De Pergola G Campobasso N Nardecchia A Triggiani V Caccavo D Gesualdo L. Para- and Perirenal Ultrasonographic Fat Thickness is Associated With 24-Hours Mean Diastolic Blood Pressure Levels in Overweight and Obese Subjects. BMC Cardiovasc Disord (2015) 15:108. 10.1186/s12872-015-0101-6 26419359 PMC4588871

[191] Fabbrini E Tamboli RA Magkos F Marks-Shulman PA Eckhauser AW Richards WO. Surgical Removal of Omental Fat Does Not Improve Insulin Sensitivity and Cardiovascular Risk Factors in Obese Adults. Gastroenterology (2010) 139(2):448–55. 10.1053/j.gastro.2010.04.056 PMC2910849 20457158

[192] Ricci MA Scavizzi M Ministrini S De Vuono S Pucci G Lupattelli G. Morbid Obesity and Hypertension: The Role of Perirenal Fat. J Clin Hypertens (Greenwich) (2018) 20(10):1430–7. 10.1111/jch.13370 PMC8030925 30216641

[193] Sahin SB Durakoglugil T Ayaz T Sahin OZ Durakoglugil E Sumer F. Evaluation of Para- and Perirenal Fat Thickness and its Association With Metabolic Disorders in Polycystic Ovary Syndrome. Endocr Pract (2015) 21(8):878–86. 10.4158/EP14435.OR 26121442

[194] Okeahialam BN Sirisena AI Ike EE Chagok NM. Ultrasound Assessed Peri-Renal Fat: An Index of Sub-Clinical Atherosclerosis. Am J Cardiovasc Dis (2020) 10(5):564–8. PMC7811913 33489459

[195] Kortelainen ML Särkioja T. Extent and Composition of Coronary Lesions and Degree of Cardiac Hypertrophy in Relation to Abdominal Fatness in Men Under 40 Years of Age. Arterioscler Thromb Vasc Biol (1997) 17(3):574–9. 10.1161/01.ATV.17.3.574 9102179

[196] Grima P Guido M Zizza A Chiavaroli R. Sonographically Measured Perirenal Fat Thickness: An Early Predictor of Atherosclerosis in HIV-1-Infected Patients Receiving Highly Active Antiretroviral Therapy? J Clin Ultrasound (2010) 38(4):190–5. 10.1002/jcu.20664 20091697

[197] Bassols J Martínez-Calcerrada JM Prats-Puig A Carreras-Badosa G Xargay-Torrent S Lizarraga-Mollinedo E. Perirenal Fat is Related to Carotid Intima-Media Thickness in Children. Int J Obes (Lond) (2018) 42(4):641–7. 10.1038/ijo.2017.236 29064476

[198] Wang D Hu B Hu C Zhu F Liu X Zhang J. Clinical Characteristics of 138 Hospitalized Patients With 2019 Novel Coronavirus-Infected Pneumonia in Wuhan, China. Jama (2020) 323(11):1061–9. 10.1001/jama.2020.1585 PMC7042881 32031570

[199] Bramlage P Pittrow D Wittchen HU Kirch W Boehler S Lehnert H. Hypertension in Overweight and Obese Primary Care Patients is Highly Prevalent and Poorly Controlled. Am J Hypertens (2004) 17(10):904–10. 10.1016/j.amjhyper.2004.05.017 15485752

[200] Xiong XQ Chen WW Zhu GQ. Adipose Afferent Reflex: Sympathetic Activation and Obesity Hypertension. Acta Physiol (Oxf) (2014) 210(3):468–78. 10.1111/apha.12182 24118791

[201] Ghaben AL Scherer PE. Adipogenesis and Metabolic Health. Nat Rev Mol Cell Biol (2019) 20(4):242–58. 10.1038/s41580-018-0093-z 30610207

[202] Libby P Buring JE Badimon L Hansson GK Deanfield J Bittencourt MS. Atherosclerosis. Nat Rev Dis Primers (2019) 5(1):56. 10.1038/s41572-019-0106-z 31420554

[203] Yang CW Guo YC Li CI Liu CS Lin CH Liu CH. Subclinical Atherosclerosis Markers of Carotid Intima-Media Thickness, Carotid Plaques, Carotid Stenosis, and Mortality in Community-Dwelling Adults. Int J Environ Res Public Health (2020) 17(13):1–14. 10.3390/ijerph17134745 PMC7369727 32630321

[204] Willeit P Tschiderer L Allara E Reuber K Seekircher L Gao L. Carotid Intima-Media Thickness Progression as Surrogate Marker for Cardiovascular Risk: Meta-Analysis of 119 Clinical Trials Involving 100 667 Patients. Circulation (2020) 142(7):621–42. 10.1161/CIRCULATIONAHA.120.046361 PMC7115957 32546049

[205] Benjamin EJ Blaha MJ Chiuve SE Cushman M Das SR Deo R. Heart Disease and Stroke Statistics-2017 Update: A Report From the American Heart Association. Circulation (2017) 135(10):e146–603. 10.1161/CIR.0000000000000491 PMC5408160 28122885

[206] Longo M Zatterale F Naderi J Parrillo L Formisano P Raciti GA. Adipose Tissue Dysfunction as Determinant of Obesity-Associated Metabolic Complications. Int J Mol Sci (2019) 20(9):1–23. 10.3390/ijms20092358 PMC6539070 31085992

[207] Tchang BG Saunders KH Igel LI. Best Practices in the Management of Overweight and Obesity. Med Clin N Am (2021) 105(1):149–74. 10.1016/j.mcna.2020.08.018 33246516

[208] Beamish AJ Olbers T Kelly AS Inge TH. Cardiovascular Effects of Bariatric Surgery. Nat Rev Cardiol (2016) 13(12):730–43. 10.1038/nrcardio.2016.162 27762312

[209] Khan T Hamilton MP Mundy DI Chua SC Scherer PE. Impact of Simvastatin on Adipose Tissue: Pleiotropic Effects *in Vivo* . Endocrinology (2009) 150(12):5262–72. 10.1210/en.2009-0603 PMC2795715 19819942

[210] Kim Y Park CW. Mechanisms of Adiponectin Action: Implication of Adiponectin Receptor Agonism in Diabetic Kidney Disease. Int J Mol Sci (2019) 20(7):1–12. 10.3390/ijms20071782 PMC6480391 30974901

[211] Tonolo G Melis MG Formato M Angius MF Carboni A Brizzi P. Additive Effects of Simvastatin Beyond its Effects on LDL Cholesterol in Hypertensive Type 2 Diabetic Patients. Eur J Clin Invest (2000) 30(11):980–7. 10.1046/j.1365-2362.2000.00735.x 11114960

[212] Sakamoto K Sakamoto T Ogawa H. The Effect of 6 Months of Treatment With Pravastatin on Serum Adiponection Concentrations in Japanese Patients With Coronary Artery Disease and Hypercholesterolemia: A Pilot Study. Clin Ther (2006) 28(7):1012–21. 10.1016/j.clinthera.2006.07.001 16990079

[213] Deng G Long Y Yu YR Li MR. Adiponectin Directly Improves Endothelial Dysfunction in Obese Rats Through the AMPK-Enos Pathway. Int J Obes (Lond) (2010) 34(1):165–71. 10.1038/ijo.2009.205 19823181

[214] Arita Y Kihara S Ouchi N Maeda K Kuriyama H Okamoto Y. Adipocyte-Derived Plasma Protein Adiponectin Acts as a Platelet-Derived Growth Factor-BB-Binding Protein and Regulates Growth Factor-Induced Common Postreceptor Signal in Vascular Smooth Muscle Cell. Circulation (2002) 105(24):2893–8. 10.1161/01.CIR.0000018622.84402.FF 12070119

[215] Wang Y Lam KS Xu JY Lu G Xu LY Cooper GJ. Adiponectin Inhibits Cell Proliferation by Interacting With Several Growth Factors in an Oligomerization-Dependent Manner. J Biol Chem (2005) 280(18):18341–7. 10.1074/jbc.M501149200 15734737

[216] Nomura S Shouzu A Omoto S Inami N Shimazu T Satoh D. Effects of Pitavastatin on Monocyte Chemoattractant Protein-1 in Hyperlipidemic Patients. Blood Coagul Fibrinolysis (2009) 20(6):440–7. 10.1097/MBC.0b013e32832e0618 19525846

[217] Wanders D Plaisance EP Judd RL. Pharmacological Effects of Lipid-Lowering Drugs on Circulating Adipokines. World J Diabetes (2010) 1(4):116–28. 10.4239/wjd.v1.i4.116 PMC3083894 21537437

[218] Okuma H Mori K Nakamura S Sekine T Ogawa Y Tsuchiya K. Ipragliflozin Ameliorates Diabetic Nephropathy Associated With Perirenal Adipose Expansion in Mice. Int J Mol Sci (2021) 22(14):7329. 10.3390/ijms22147329 34298949 PMC8304702

[219] Packer M. Mitigation of the Adverse Consequences of Nutrient Excess on the Kidney: A Unified Hypothesis to Explain the Renoprotective Effects of Sodium-Glucose Cotransporter 2 Inhibitors. Am J Nephrol (2020) 51(4):289–93. 10.1159/000506534 32126558

[220] Morano S Romagnoli E Filardi T Nieddu L Mandosi E Fallarino M. Short-Term Effects of Glucagon-Like Peptide 1 (GLP-1) Receptor Agonists on Fat Distribution in Patients With Type 2 Diabetes Mellitus: An Ultrasonography Study. Acta Diabetologica (2015) 52(4):727–32. 10.1007/s00592-014-0710-z 25577244

[221] Chen J Zhao H Ma X Zhang Y Lu S Wang Y. GLP-1/GLP-1R Signaling in Regulation of Adipocyte Differentiation and Lipogenesis. Cell Physiol Biochem (2017) 42(3):1165–76. 10.1159/000478872 28668964

